# Genome Mining for Diazo-Synthesis-Related Genes in *Streptomyces* sp. CS057 Unveiled the Cryptic Biosynthetic Gene Cluster *crx* for the Novel 3,4-AHBA-Derived Compound Crexazone 2

**DOI:** 10.3390/biom14091084

**Published:** 2024-08-29

**Authors:** Laura Prado-Alonso, Suhui Ye, Ignacio Pérez-Victoria, Ignacio Montero, Pedro Riesco, Francisco Javier Ortiz-López, Jesús Martín, Carlos Olano, Fernando Reyes, Carmen Méndez

**Affiliations:** 1 Departamento de Biología Funcional e Instituto Universitario de Oncología del Principado de Asturias (IUOPA), Universidad de Oviedo, 33006 Oviedo, Spain; pradoalaura@uniovi.es (L.P.-A.); yesuhui@uniovi.es (S.Y.); nachomontero@gmail.com (I.M.); pedro.riesco@ipla.csic.es (P.R.); olanocarlos@uniovi.es (C.O.); 2 Instituto de Investigación Sanitaria de Asturias (ISPA), 33011 Oviedo, Spain; 3 Fundación MEDINA, Centro de Excelencia en Investigación de Medicamentos Innovadores en Andalucía, Armilla, 18016 Granda, Spain; ignacio.perez-victoria@medinaandalucia.es (I.P.-V.); javier.ortiz@medinaandalucia.es (F.J.O.-L.); jesus.martin@medinaandalucia.es (J.M.); fernando.reyes@medinaandalucia.es (F.R.)

**Keywords:** *Streptomyces*, *N-N* bond, 3,4-AHBA, diazo, genome mining, secondary metabolites, natural products, biosynthetic gene clusters

## Abstract

Natural products play a crucial role in drug development, addressing the escalating microbial resistance to antibiotics and the treatment of emerging diseases. Progress in genome sequencing techniques, coupled with the development of bioinformatics tools and the exploration of uncharted habitats, has highlighted the biosynthetic potential of actinomycetes. By in silico screening for diazo-related gene genomes from twelve *Streptomyces* strains isolated from *Attini* leaf-cutting ants, the new *crx* biosynthetic gene cluster (BGC) was identified in *Streptomyces* sp. CS057. This cluster, highly conserved in several *Streptomyces* strains, contains genes related to diazo group formation and genes for the biosynthesis of 3,4-AHBA. By overexpressing the LuxR-like regulatory gene *crxR1*, we were able to activate the *crx* cluster, which encodes the biosynthesis of three 3,4-AHBA-derived compounds that we named crexazones (CRXs). The chemical structure of crexazones (CRXs) was determined by LC-DAD-HRMS-based dereplication and NMR spectroscopic analyses and was found to correspond to two known compounds, 3-acetamido-4-hydroxybenzoic acid (CRX1) and the phenoxazinone texazone (CRX3), and a novel 3,4-AHBA-containing compound herein designated as CRX2. Experimental proof linking the *crx* BGC to their encoded compounds was achieved by generating mutants in selected *crx* genes.

## 1. Introduction

The discovery of new drugs to counteract the growing antibiotic resistance and to treat known and emerging diseases for which an appropriate treatment does not exist is one of the challenges humanity faces today. Natural products (NPs) and their derivatives have historically been the primary contributors to the arsenal of drugs available on the market [1].

The phylum Actinobacteria comprises a group of Gram-positive bacteria with high G+C content, most of which exhibit complex morphological differentiation cycles, inhabit various environments, and have a significant portion of their genome dedicated to secondary metabolism. As such, they are traditionally known to be producers of a wide variety of natural products with diverse bioactivities and applications in healthcare, biotechnology, or environmental sectors [2,3]. Among Actinobacteria, *Streptomyces* has been and continues to be the most prolific genus for bioactive secondary metabolite production, being the source of the majority of clinically used natural antibiotics [3,4,5]. However, limitations in screening methods and the depletion of traditional ecological niches have generated the need to develop new strategies and solutions to these challenges and to find new environmental niches [6]. In this regard, the improvement of genome sequencing technologies, bioinformatics tools, and search methods such as genome mining have highlighted the great biosynthetic potential of actinomycetes due to their high content of unknown or cryptic biosynthetic gene clusters (BGCs) [7]. These methods, in combination with the exploration of new environments such as the ocean [8], extreme environments [9], or in association with other organisms [10], represent promising strategies for discovering new drugs.

To screen through this variety of BGCs, several approaches can be employed, such as the search for genes encoding novel enzymatic activities leading to novel structural groups that confer high structural and bioactivity diversity. In this sense, compounds containing *N-N* bonds constitute a structurally diverse small group of compounds displaying different biological activities that contain a variety of functional groups such as diazo, hydrazine, azoxy, piperazic acid, or N-nitroso groups [11,12,13]. Their biosynthetic processes are diverse and still poorly understood, although the discovery in recent decades of different types of enzymes involved in *N-N* bond formation has shed new light on perspectives [14]. (Figure S1): compound S56-P1 is a hydrazone-containing compound that is synthesized through a hydrazine intermediate. This is synthesized by the Spb38 lysine N-hydroxylase that hydroxylates L-lysine and the didomain fusion protein Spb40, which contains a C-terminal domain similar to methionyl-tRNA synthetases and an N-terminal domain that belongs to the cupin superfamily. Spb40 synthesizes an intermediate from N-hydroxy-L-Lysine and glycine that, after suffering a rearrangement, generates the *N-N* bond of the hydrazine intermediate. Formation of the N-nitroso group in streptozocin proceeds by methylating L-arginine by the StzE/SznE N-methyltransferase, followed by the action of StzF/SznF. This is a didomain protein with an N-terminal diiron domain and a C-terminal cupin domain. The diiron domain catalyzes the dihydroxylation of the methylated arginine, which undergoes an oxidative rearrangement by the cupin domain, generating an N-nitroso-contained intermediate; the azoxy group in azoxymicins is supposed to derive from either a nitroso or a hydroxylamine compound, which are synthesized by the nonheme diiron-dependent N-oxidase AzoC; the biosynthesis of piperazic acid in kutzneride involves the KtzI ornithine N-hydroxylase for hydroxylating L-ornithine and the KtzT piperazate synthase for *N-N* bond formation in the hydroxylated ornithine. Regarding diazo groups in cremeomycin, the nitrogen contributing to their formation is provided by nitrite or nitrous acid, which is synthesized through the aspartate-nitro-succinate (ANS) pathway via the sequential action of two enzymes, the flavin monooxygenase CreE, and the 3-carboxy-*cis*,*cis*-muconate cycloisomerase CreD [15]. In addition, subsequent action of the CreM AMP-dependent ligase catalyzing diazotization is required [16]. Several compounds with diazo groups have been described where CreD and CreE similar enzymes synthesize nitrous acid, and CreM-like ligases carry out diazo group formation: spinamycin (SpiD, SpiE, and SpiA7) [17]; alazopeptin (AzpD, AzpE, and AzpL) [18]; tasikamydes (Aha1, Aha2, and Aha11) [19]; avenalumic acid (AvaD, AvaE, and AvaA6) [20]; or triacsin (Tri16, Tri21, and Tri17) [21].

The symbiosis between actinomycetes and insects has recently emerged as a novel niche for the discovery of new compounds [10,22,23,24]. Studies carried out in our research group have previously revealed the potential of the “CS collection,” a collection of Actinomycetes strains isolated from *Attini* leaf-cutting ants, for the discovery of new natural products [13,25,26,27,28,29]. In this work, we report a screening of genes related to the biosynthesis of *N-N* bonds in 12 *Streptomyces* strains belonging to the “CS collection” whose genomes have been previously sequenced. This analysis revealed the existence of the *crx* BGC in *Streptomyces* sp. CS057, which contains genes encoding similar proteins to those responsible for the biosynthesis of the diazo group in cremeomycin [15]. We also show the activation of this cluster by overexpressing a LuxR regulatory gene and the identification of its encoded compounds that derive from 3-amino-4-hydroxybenzoic acid (3,4-AHBA), one of which is a novel compound named crexazone 2 (CRX 2).

## 2. Materials and Methods

### 2.1. Strains and Culture Conditions, Plasmids, and DNA Manipulations

Bioinformatics screening analyses were performed on genomes from the following *Streptomyces* strains belonging to the “CS collection” [25]: *Streptomyces* sp. CS090 (NZ_QBHY00000000.1), CS113 (NZ_NEVC00000000.1), CS159 (NZ_NEVD00000000.1), CS014 (NZ_QBHV00000000.1), CS057 (NZ_NEVF00000000.1), CS147 (NZ_QBIA00000000.1), CS131 (NZ_QBHZ00000000.1), CS149 (NZ_PVZY00000000.1), CS065a (NZ_QBHW00000000.1), CS207 (NZ_QBIB00000000.1), CS227 (NZ_NEVE00000000.1, PRJNA384387), and CS081a (NZ_QBHX00000000.1). *Streptomyces* sp. CS057 was used as a source of DNA to construct plasmids and to generate mutants. *Escherichia coli* DH10B (Invitrogen, Waltham, MA, USA) was used for cloning, while *Escherichia coli* ET12567/pUZ8002 [30] was used for intergeneric conjugation between *E. coli* and *Streptomyces* sp.

MA medium was utilized for the sporulation of *Streptomyces* strains [31]. The conjugation experiments were carried out following a described procedure [30] using MS medium [32]. When necessary, antibiotics were added to the culture medium for selection at the following final concentrations: apramycin (100 µg/mL for *E. coli* and 50 µg/mL for *Streptomyces*); kanamycin (25 µg/mL for *E. coli* and 200 µg/mL for *Streptomyces*); chloramphenicol (25 µg/mL); ampicillin (100 µg/mL); hygromycin (200 µg/mL); thiostrepton (25 µg/mL in solid medium and 5 µg/mL in liquid medium); and nalidixic acid (50 µg/mL).

For secondary metabolite production, 250 mL baffled flasks containing 50 mL of Tryptic Soy Broth (TSB, Oxoid) were inoculated with *Streptomyces* spores and incubated for two days at 30 °C and 250 rpm. Then, these cultures were used to inoculate 250 mL amber flasks containing 50 mL of R5A medium [33] at a final optical density at 600 nm (OD600) of 0.2, and cultures were incubated at 30 °C and 250 rpm for 6 days.

DNA manipulation was carried out following standard procedures for *E. coli* [34] and *Streptomyces* [30]. Chromosomal DNA extraction for PCR was performed using the phenol-chloroform-isoamyl alcohol protocol [30]. DNA amplifications to construct plasmids were carried out using Herculase II Fusion High-Fidelity Polymerase (Invitrogen, Waltham, MA, USA) following the manufacturer’s instructions. DNA amplifications for plasmid construction and mutant verifications were performed using DreamTaq Polymerase (Thermo Fisher Scientific, Waltham, MA, USA) following the supplier’s instructions. DNA amplifications for mutant and recombinant strain verification via *Streptomyces* colony PCR were carried out using Terra Polymerase (Takara, Kusatsu, Japan) following the manufacturer’s instructions.

Several plasmid vectors were used along this work: pCRBlunt (Invitrogen, Waltham, MA, USA) for DNA cloning; pUO9090 [35] as the source of the apramycin resistance cassette; pHZ1358 [36] for gene replacement; pSETEcH [29] and pOJ260p [37] for gene overexpression in *Streptomyces* using the ermE* promoter; and pSETxk [38] for gene overexpression in *Streptomyces* using the kasO* promoter. BLAST [39] was used to compare genes and proteins, and antiSMASH 7.1.0 was used to analyze BGCs and non-ribosomal peptide synthetase (NRPS) and polyketide synthase (PKS) domains [40].

### 2.2. Generation of Mutants

Several mutants were generated (Table 1) by replacing specific genes with an apramycin resistance cassette. These mutants were genetically confirmed by PCR and by sequencing the PCR products using oligonucleotides from Table S1. To this end, several plasmid constructs were generated (Table 1).

#### 2.2.1. Generation of ΔAHBA Mutant

This mutant was constructed using plasmid pHZ1358ΔAHBA as follows: First, a 2200-bp fragment (fragment 1) containing *crxR1* was amplified using oligonucleotides Mut1A_BglII and Mut1B_EcoRI, and a 2000-bp fragment (fragment 2) containing *crxA1* to *crxB* was amplified using oligonucleotides Mut2ABglII and Mut2BEcoRV. After independently cloning these fragments into pCRBlunt, fragment 1 was released with BglII and EcoRI and cloned into the same sites of pUO9090. Subsequently, fragment 2 was released with XbaI and SpeI and cloned into the XbaI site of pUO9090+fragment1. Finally, a 4500-bp fragment containing fragment1-apramycin-fragment 2 was obtained by digestion with SpeI and subcloned into the XbaI site of pHZ1358.

#### 2.2.2. Generation of Mutants by Gene Replacement Using Gibson Assembly Plasmids

Mutants ΔNRPS, Δ*crxA1*, Δ*crxC*, Δ*crxD*, Δ*crxE*, Δ*crxM*, and ΔTetR were generated using pHZ1358-derived plasmids, which were constructed by Gibson assembly as previously described in [13].

- pHZ1358ΔNRPS was used to delete *orf4*, generating the ΔNRPS mutant. It was constructed by Gibson assembly between pHZ1358 digested with NheI and XbaI; fragment A (2095 bp), containing the 5′-end of *orf4*, *orf5,* and *orf6* and amplified with oligonucleotides AdNRPS FW and RV; fragment B (2554 bp), containing the 3′-end of *orf4*, and *orf1* to *orf3*, amplified with oligonucleotides BdNRPS FW and RV; and the apramycin cassette, amplified from pUO9090 with oligonucleotides ApraGibsFW and RV.
- pHZ1358Δ11 was used to delete *crxA1*, generating the Δ*crxA1* mutant. Fragment A (2337 bp), containing *orf10* and the 5′-end of *crxA1*, was amplified with oligonucleotides d11AFW and RV; fragment B (2627 bp), containing the 3′-end of *crxA1*, *crxA2*, *crxB*, and the 3′-end of *crxC*, was amplified with oligonucleotides d11BFW and RV.
- pHZ1358Δ14 was used to delete *crxC*, generating the Δ*crxC* mutant. Fragment A (2181 bp), containing the 5′-end of *crxC* and *crxA3*, was amplified with oligonucleotides d14AFW and RV; fragment B (3662 bp), containing the 3′-end of *crxC* and *crxA1* to *crxB*, was amplified with oligonucleotides d14BFW and RV.
- pHZ1358Δ16 was used to delete *crxD*, generating the Δ*crxD* mutant. Fragment A (2512 bp), containing *crxE*, was amplified with oligonucleotides d16AFW and RV; fragment B (2655 bp), containing the 3′-end of *crxD*, *crxA3*, and the 5′-end of *crxC*, was amplified with oligonucleotides d16BFW and RV.
- pHZ1358Δ17 was used to delete *crxE*, generating the Δ*crxE* mutant. Fragment A (1993 bp), containing the 5′-end of *crxE*, *crxK,* and *crxI*, was amplified with oligonucleotides d17AFW and RV; fragment B (2343 bp), containing the 5′-end of *crxA3*, *crxD*, and the 3′-end of *crxE*, was amplified with oligonucleotides d17BFW and RV.
- pHZ1358Δ22 was used to delete *crxM*, generating the Δ*crxM* mutant. Fragment A (3035 bp), containing *crxR1*, *crxF*, and the 5′-end of *crxM*, was amplified with oligonucleotides d22AFW and RV; fragment B (2907 bp), conatining the 3′-end of *crxM*, *crxN,* and the 3′-end of *crxH*, was amplified with oligonucleotides d22BFW and RV.
- pHZ1358ΔTetR was used to delete *crxR2*, generating the ΔTetR mutant. Fragment A (2600 bp), containing the 5′-end of *crxR1*, *crxT*, *crxP*, and the 5′-end of *crxR2*, was amplified with oligonucleotides dTetRAFW and RV; fragment B (2700 bp), containing the 3′-end of *crxR2*, *orf28* and the 3′-end of *orf29*, was amplified with oligonucleotides dTetRBFW and RV.

### 2.3. Generation of Recombinant Strains

Several recombinant strains were generated by expressing *crx* genes into CS057 wild-type (WT) and/or mutant strains (Table 1). For this purpose, different plasmids were constructed using oligonucleotides from Table S1.

- pSETEcHLuxR24 was constructed to overexpress *crxR1,* encoding a LuxR regulator under the control of the ermE*p promoter. *CrxR1* (2539 bp) was amplified with the oligonucleotides eLuxR24XbaIFW and eLuxR24EcoRVRV and cloned into the XbaI and EcoRV sites of pSETEcH.
- pSETEcHLuxRA was constructed to overexpress the LuxR regulator encoded by *orfA* under the control of the ermE*p promoter. *orfA* (1049 bp), amplified with the oligonucleotides eLuxRAEcoRVFW and eLuxRAEcoRVRV, and cloned into the EcoRV site of pSETEcH.
- pSETEcHLuxR24A was constructed to overexpress both LuxR regulators encoded by *orfA* and *crxR1* under the control of the ermE*p promoter *orfA* (1049 bp), amplified with the oligonucleotides orfApSETHGibsFWXbaI and orfApSETHGibsRVcorf24 and *crxR1* (2539 bp), and amplified with oligonucleotides orf24FW and orf24gibsRVEcoRV. Both fragments were then cloned by Gibson assembly into pSETEcH digested with XbaI and EcoRV.
- pSETxkLuxR24 was constructed to overexpress *crxR1* under the control of the kasOp* promoter. *CrxR1* (2612 bp) was amplified with oligonucleotides FW24c3pSETxkBamHI and RV24c5pSETxkEcoRV and cloned by Gibson assembly into pSETxk digested with BamHI and EcoRV.
- pOJ260pLuxR24 was constructed to insert the ermE*p promoter upstream *crxR1. CrxR1* (2539 bp) was amplified with oligonucleotides eLuxR24XbaIFW and eLuxR24EcoRVRV and cloned into the XbaI and EcoRV sites of pOJ260p.
- pSETxKTetR was constructed to overexpress the TetR regulator encoded by *crxR2* under the control of the kasOp* promoter. *CrxR2* (770 bp), amplified with the oligonucleotides kTetRcpSETxkBamHIFW and kTetRcpSETxkEcoRVRV, and cloned by Gibson assembly into pSETxk, digested with BamHI and EcoRV.
- pOJ260pNRPS was constructed to insert the ermE*p promoter upstream of the NRPS-coding gene *orf4*. This plasmid was generated by cloning a 5′-end fragment of *orf4* amplified by PCR with the oligonucleotides pNRPSXbaIFW and pNRPSEcoRIRV into the XbaI and EcoRI sites of pOJ260p.
- pSETEcHc11LuxR24 was constructed to complement the Δ*crxA*1 mutant. *CrxA1* (1200 bp) was amplified with the oligonucleotides cORF11Lux24c5Lux24EcoRVFW and cORF11Lux24c5pSETHEcoRVRV and cloned by Gibson assembly into pSETEcHLuxR24 digested with EcoRV downstream of *crxR1*.

### 2.4. Chromatografic Analysis, Isolation and Structural Determination of Crexazones

#### 2.4.1. Ultraperformance Liquid Chromatography (UPLC)

All experiments for the production and extraction of secondary metabolites were conducted under dark conditions, using amber flasks and tubes. R5A cultures after 6 days of growth were extracted with one volume of ethyl acetate containing 1% formic acid for 1 h at room temperature. The organic phases were dried down and resuspended in 75 µL of methanol. Samples were analyzed by UPLC using an Acquity UPLC I-class system (Waters, Milford, MA, USA) with an injected volume of 10 µL. BEH C18 column (1.7 µm, 2.1 × 100 mm; Waters, Milford, MA, USA) was routinely used, and HSS T3 column (1.8 μm, 2.1 × 100 mm; Waters, Milford, MA, USA) was utilized to enhance the analysis of polar and hydrophobic analytes if necessary. The mobile phase consisted of solvent A (acetonitrile) and solvent B (Milli-Q water with 0.1% trifluoroacetic acid, TFA). An elution gradient was established over 10 min from 10 to 100% acetonitrile and from 90 to 0% Milli-Q water with 0.1% TFA at a flow rate of 0.5 mL/min. As the compounds absorb at several wavelengths, UPLC-UV Max Plot chromatograms (ranging from 210 nm to 410 nm) were used by default, and a wavelength of λ 400 nm was used to specifically analyze peaks with compounds CRX2 and CRX3.

#### 2.4.2. Isolation of Crexazones and Compound Dereplication Based on LC-DAD-HRMS Analyses

Purification of compounds was carried out using the Alliance HPLC Chromatographic System (Waters, Milford, MA, USA) equipped with an Atlantis dC18 OBD Prep Column 100 Å (10 µm, 10 × 150 mm), with a mobile phase consisting of solvents A (acetonitrile) and B (MilliQ-TFA 0.1%), at a flow rate of 5 mL/min and a volume of 100 µL per injection. For the purification of compound CRX1, WT-eLuxR was cultivated in five 2 L Erlenmeyer flasks, each containing 400 mL of R5A. After 6 days of incubation in darkness, cultures were extracted with ethyl acetate supplemented with 1% formic acid for 1 h and centrifuged to collect the organic phase, which was then dried down under vacuum in a Rotavapor. The residue was resuspended in 2 mL of methanol, centrifuged again, and filtered to remove impurities. An HPLC gradient of 10% to 40% of solvent A for 60 min was employed. The fraction containing the compound was lyophilized. For the purification of CRX2 and CRX3, cultures were performed as mentioned above, but in this case they were centrifuged followed by filtration of the supernatant, which was applied to a solid-phase extraction cartridge (Sep-Pak Vac C18, 10 g, Waters, Milford, MA, USA). The retained material was eluted with a gradient from 0 to 100% of A for 55 min. Different fractions containing the different peaks of interest were collected, dried down, and purified by HPLC in isocratic conditions at 17% of A (CRX2) or 20% of A (CRX3). Fractions containing the peaks of interest were collected and lyophilized.

To determine the chemical identity of each crexazone, the compounds were first submitted to LC-DAD-HRMS-based dereplication against our in-house library [41]. Analyses were carried out using an Agilent 1200 Rapid Resolution HPLC system equipped with a SB-C8 column (2.1 × 30 mm, Zorbax, Agilent Technologies Inc, Santa Clara, CA, USA) and coupled to a Bruker maXis mass spectrometer (Bruker, Billerica, MA, USA). Chromatographic and ionization conditions were identical to those previously described [42]. Both UV/vis (DAD) and HRMS spectra for each crexazone were collected in such chromatographic analyses. Additionally, compound dereplication was approached by querying each determined molecular formula (based on the observed experimentally accurate mass) in the Dictionary of Natural Products [43].

#### 2.4.3. Structural Elucidation of Crexazone 2 (CRX2)

The structure of novel CRX2 was determined based on its molecular formula, obtained from ESI-TOF high-resolution mass spectrometry, alongside 1D and 2D NMR spectroscopic analysis. The HRMS spectrum was retrieved from the corresponding LC-DAD-HRMS analysis. NMR spectra were recorded in DMSO-d_6_ at 24 °C on a Bruker AVANCE III-500 (500 MHz and 125 MHz for ^1^H and ^13^C NMR, respectively) equipped with a 1.7 mm TCI MicroCryoProbe^TM^ (Bruker Biospin, Fällanden, Switzerland), using the residual solvent signal as internal reference (δ_H_ 2.51 and δ_C_ 40.0). Crexazone 2 (CRX2): UV (DAD): λ_max_ 245, 348 nm; ESI(+)-TOF MS *m*/*z* 289.0821 [M + H]+ (calcd. for C_14_H_13_N_2_O_5_+, 289.0819); ^1^H and ^13^C NMR data, see Table 2.

### 2.5. In Vitro Bioactivity Tests

The bioactivity of CRX2 was tested by disc diffusion assays against fungi (*Escovopsis* sp. and *Candida albicans*), Gram-positive bacteria (*Staphylococcus aureus* and *Micrococcus luteus*), and Gram-negative bacteria (*E. coli* and *Pseudomonas aeruginosa*) as previously described [29].

## 3. Results

### 3.1. Genome Mining of Streptomyces Strains for N-N Bond Formation Coding Genes

Genomes from 12 *Streptomyces* strains belonging to the “CS collection” were analyzed for the presence of genes coding for *N-N* bond formation using BlastP analysis. The following proteins were used as probes: for diazo groups, CreD, and CreE responsible for nitrous acid biosynthesis in cremeomycin [15,16,44]; for azoxy groups, AzoC from the azoxymycin BGC [45] and VlmB from the valanimycin BGC [46]; for N-nitroso groups, SznF from the streptozotocin BGC [47]; and for hydrazine groups, Spb39 and Spb40 from S56-p1 BGC [48] (Figure S1). This analysis resulted in the identification of a protein similar to AzoC in CS147 (WP_098899150.1; 61.26% identical amino acids) and several proteins similar to CreD and CreE in CS014, CS057, CS065a, CS131, CS147, CS149, and CS207, with percentages of identical amino acids ranging from 56 to 69 for CreD-homologs and from 54 to 62 for CreE-homologs (Table S2). Subsequently, antiSMASH analyses were performed to locate those coding genes at BGCs in the different strains. All identified genes were located outside BGCs except the *creD* and *creE* homologs in CS057, which were located in region 28. This region is assigned by antiSMASH as a NRPS-type gene cluster of 52.6 kb (nucleotides 6134699 to 6187252 in NZ_NEVF00000000.1).

### 3.2. Analysis of the NRPS Cluster in Region 28 from Streptomyces CS057

**Figure 1:**
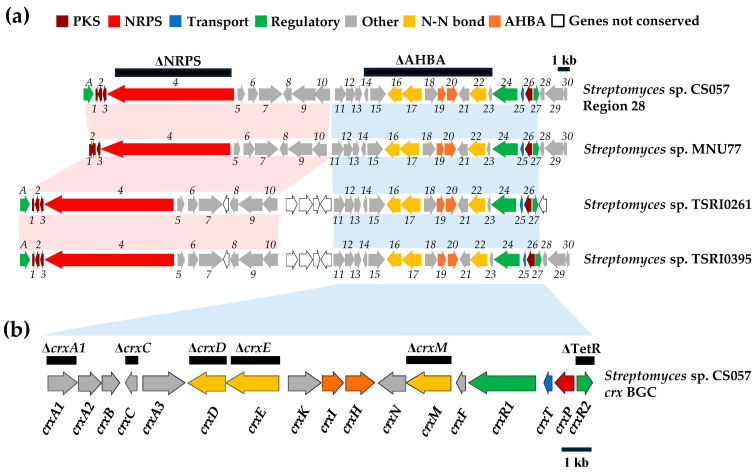
Genetic organization of region 28 from *Streptomyces* sp. CS057 and its downstream region. (**a**) Comparison to homologous gene clusters in other *Streptomyces* strains. Pink and blue bars between clusters indicate homologous genes in the “NRPS” and “AHBA” regions, respectively. (**b**) *crx* BGC. Genes are shown to scale. Black bars indicate regions that have been deleted in CS057.

The “NRPS cluster” from region 28 contains *orfA* for a transcriptional regulator: *orf1*, *orf2*, and *orf3*, for PKS-related proteins, encoding an ACP, and putative dehydratases, respectively [49]; *orf16* and *orf17* for proteins similar to CreD and CreE involved in the biosynthesis of diazo groups [15]; and *orf4* coding for an NRPS [50,51,52] (Table S3; Figure 1). Bioinformatic analyses [40] reveal that the NRPS Orf4 would be composed of 4 modules with the typical three domains for an NRPS module: a condensation (C), an adenylation (A), and a peptidyl carrier protein (PCP) domain. According to the analysis of the different conserved motifs in each domain, the NRPS would be active. The C domain from the first module corresponds to a Cstarter-type (C_S_) involved in the acylation of the first amino acid of a peptide chain. The remaining C domains are of the ^L^C_L_ type and perform the condensation of two L-amino acids. The specificity of the A domains for specific amino acids was in silico determined through bioinformatics analyses using antiSMASH [40] and by comparing the identified binding pockets with those reported in the literature [50,51]. As a result, Orf4 is predicted to synthesize a tetrapeptide constituted by Thr-DhpG-Asn/Asp-Gly. The NRPS does not contain a thioesterase domain.

Further analysis of the NRPS cluster downstream region unveiled the presence of additional genes, some of which code for proteins frequently identified in other BGCs, such as a LuxR-type regulator (*orf24*), a TetR-type repressor (*orf27*), or an MFS transporter (*orf25*). Additionally, this region also contains genes coding for similar proteins to CreM ligase (*orf22*), crucial for the formation of the diazo group in cremeomycin [16]; to CreN SAM-dependent methyltransferase (*orf21*), that incorporates a methyl group in cremeomycin [16,44]; and to CreH and CreI (*orf19*, *orf20*), responsible for catalyzing the biosynthesis of 3-amino-4-hydroxybenzoic acid (3,4-AHBA) [44].

BlastP analyses of these Orfs in region 28 and of those located in the adjacent downstream region allowed for the identification of similar BGCs in different *Streptomyces* strains with which they share high synteny (Figure 1a, Table S3). From this analysis, a preliminary delimitation of the cluster was proposed, which would encompass two highly conserved regions. An “NRPS region” between *orfA* and *orf10* would include the NRPS *orf4*, the PKS-related *orf1*, *orf2*, and *orf3* genes, and the LuxR regulator *orfA*. This region could encode a peptidic compound with a carbon chain attached. The second conserved region (AHBA region), between *orf11* and *orf27*, contains genes related to the biosynthesis of 3,4-AHBA (*orf19*, *orf20*), diazo group formation (*orf16*, *orf17*, *orf22*), as well as genes encoding the LuxR and TetR-type regulators (*orf24*, *orf27*), and an MFS transporter (*orf25*). These genes would encode a 3,4-AHBA-derived compound. From this analysis, it is not clear whether these two regions constitute a single cluster or two different clusters. As shown in Figure 1a, there are several *Streptomyces* strains that contain these two regions, displaying the same genetic organization and only differing in a few additional genes that are normally inserted between both regions. Noteworthy, other *Streptomyces* strains contain the “AHBA region” but not the “NRPS region”.

### 3.3. The crx Biosynthetic Gene Cluster and Its Encoded Compounds

To identify the compound(s) encoded by region 28 and to determine if the “NRPS” and “AHBA” regions are part of a single cluster or two different clusters, two independent mutants were generated in these regions (Table 1; Figure 1a). The ΔAHBA mutant is affected in the “AHBA region” and was constructed using plasmid pHZ1358ΔAHBA (Table 1) by deleting *orf14* to *orf23* and replacing them by the apramycin resistance gene. The genotype of the resultant mutant strain was confirmed by PCR (Figure S2): using oligonucleotides CS057MutCA/ApraCRV and CS057MutCB/ApraCFW (Table S1), a 2573 bp DNA fragment A and a 2129 bp DNA fragment B were amplified from ΔAHBA and not from the WT strain. Regarding the “NRPS region”, the ΔNRPS mutant was constructed by replacing *orf4* by the apramycin resistance cassette using plasmid pHZ1358ΔNRPS (Table 1). Its genotype was confirmed by PCR (Figure S3): using oligonucleotides dNRPSIFW/ApraI and dNRPSDRV/ApraD (Table S1), a 2600 bp fragment A and a 3200 bp fragment B were amplified by the mutants. These fragments were not amplified from the WT strain.

The metabolite profiles of the wild-type and ΔAHBA and ΔNRPS mutants were compared using UPLC (Figure S4). No differences were observed between the WT and the mutant strains, except for a peak (Figure S4) detected in the WT and in the ΔAHBA mutant but not in ΔNRPS. This initially suggested that this compound could be codified by the “NRPS region.” However, further LC-HRMS analysis of this peak showed it had a *m*/*z* value of 282 and a molecular formula of C_15_H_23_NO_4_, which did not fit with the predicted mass for the peptidic compound synthesized by Orf4 NRPS. Therefore, these results indicate that region 28 must be silent or poorly expressed under the culture conditions tested.

**Figure 2:**
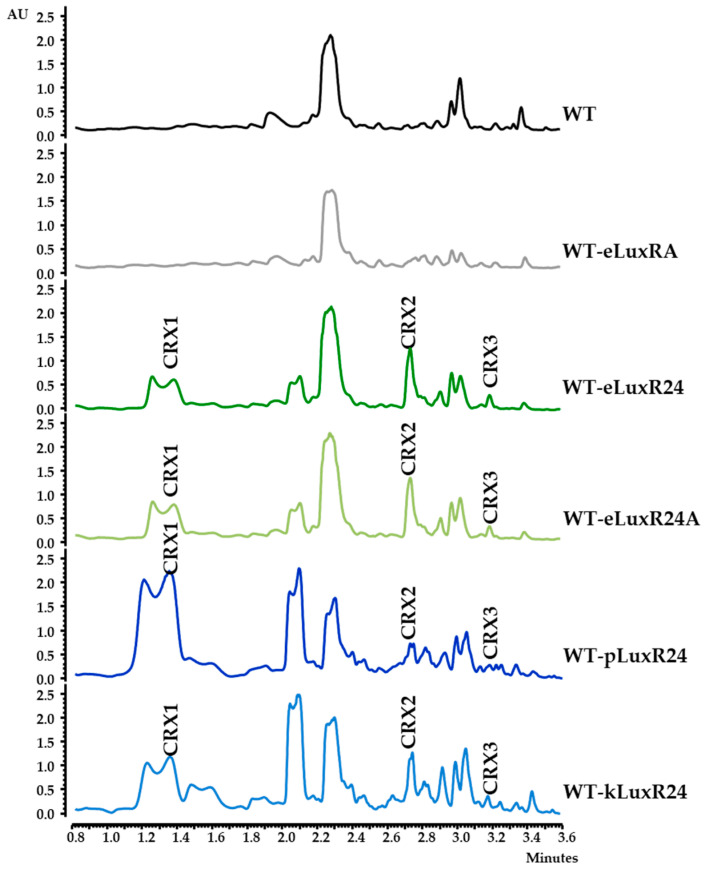
Effect of overexpressing LuxR regulators on the production of crexazones in *Streptomyces* CS057. UPLC Max Plot chromatograms of ethyl acetate-1% formic acid extracts of cultures in R5A medium in dark conditions. CRX, crexazone; WT, wild type; WT-eLuxRA, WT overexpressing *orfA*; WT-eLuxR24, WT overexpressing *orf24* (*crxR1*); WT-eLuxR24A, WT overexpressing *orfA* and *orf24* (*crxR1*); WT-pLuxR24, WT with the ermE*p promoter inserted upstream of *orf24* (*crxR1*)*;* WT-kLuxR24, WT overexpressing *orf24* (*crxR1*) under the control of the kasOp* promoter.

To activate the expression of region 28, several strategies were followed:

(i) Inactivation/overexpression of *crxR2*. Region 28 contains the TetR-like regulatory gene *crxR2* (*orf27*) (Figure 1). TetR regulators usually act as repressors, although some can act as activators [53,54]. To evaluate its putative role in region 28, *crxR2* was deleted and replaced by the apramycin resistance gene using plasmid pHZ1358ΔTetR (Table 1). The resultant mutant ΔTetR was genetically confirmed by PCR: using oligonucleotides dTetRIFW/ApraI and dTetRDRV/ApraD (Table S1), a 2600 bp fragment A and a 2700 bp fragment B were amplified from the mutant and not from the WT strain (Figure S5). Additionally, *crxR2* was also overexpressed in the WT strain using pSETxkTetR (Table 1). The metabolite profiles of the resultant recombinant strain WT-kTetR and of the ΔTetR mutant were compared to those of the WT (Figure S6). No differences were observed, indicating that the TetR regulator CrxR2 is not involved in the regulation of region 28 under the tested conditions.
(ii) Overexpression of *orfA* and/or *crxR1*. Region 28 contains two *orfs* encoding LuxR-type activators, *orfA* located at the “NRPS region” and *crxR1* (*orf24*) at the “AHBA region” (Figure 1a). These two genes were individually overexpressed in the WT strain. The resultant recombinant strains WT-eLuxRA and WT-eLuxR24 (Table 1) were cultivated in R5A medium for 6 days, and their metabolite profiles were compared with those of the WT strain. Expression of *orfA* did not lead to the production of additional compounds (Figure 2). However, upon expressing *crxR1*, a series of differential peaks were detected (Figure 2). Compounds in these peaks were named crexazones (CRX) (see below). Co-expression of *orfA* and *crxR1* (strain WT-eLuxR24A; Table 1) did not result in the production of additional peaks compared to those produced by the WT-eLuxR24 strain (Figure 2). These results indicated that the production of those differential peaks only requires the expression of *crxR1*. To evaluate a potential improvement in the production of those compounds, the ermE*p promoter was inserted upstream of *crxR1* in the chromosome of WT using plasmid pOJ260pLuxR24 (Table 1). The right insertion of this promoter in the resultant recombinant strain WT-pLuxR24 was confirmed by PCR using oligonucleotides M13FW and eLuxR24DRV (Table S1), which amplified a 3400 bp DNA fragment from the recombinant strain and not from the WT strain (Figure S7). Moreover, *crxR1* was overexpressed *in trans* under the control of the kasOp* promoter, using plasmid pSETxkLuxR24 (Table 1), generating the WT-kLuxR24 strain. Culture extracts from both strains did not show any additional differential peaks. However, some of the already detected compounds were produced in higher amounts: compounds CRX1 and CRX2 in WT-kLuxR24 and CRX1 in WT-pLuxR24 (Figure 2).

**Figure 3:**
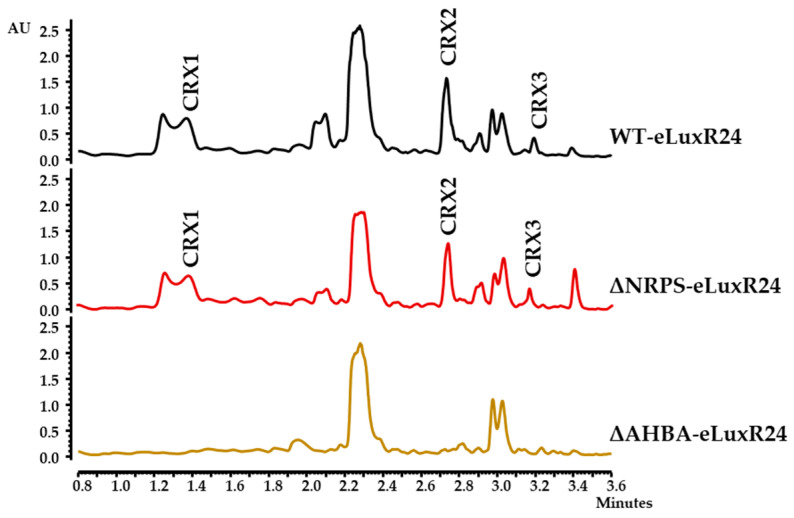
Production of crexazones by *Streptomyces* sp. CS057. UPLC chromatograms (Max Plot) of ethyl acetate-formic acid extracts of *Streptomyces* sp. WT-eLuxR24 and mutants ΔNRPS-eLuxR24 and ΔAHBA-eLuxR24 cultivated in R5A medium. WT-eLuxR24, wild-type overexpressing *orf24* (*crxR1*); ΔNRPS-eLuxR24, ΔNRPS mutant overexpressing *orf24* (*crxR1*); ΔAHBA-eLuxR24, ΔAHBA mutant overexpressing *orf24* (*crxR1*); CRX, crexazone.

To confirm the involvement of region 28 in the production of those differential CRX compounds, *crxR1* was then overexpressed in the ΔAHBA and ΔNRPS mutant strains. The metabolite production profiles of the resultant strains ΔAHBA-eLuxR24 and ΔNRPS-eLuxR24 were compared to those of the WT-eLuxR24. The production of all these CRX compounds was not detected in ΔAHBA-eLuxR24. In contrast, they were still observed in the mutant ΔNRPS-eLuxR24 (Figure 3). These results suggested that production of these compounds did not require the participation of the Orf4 NRPS. To increase expression of *orf4* and see if additional compounds could be detected, which would suggest a relationship between the “NRPS” and the “AHBA” regions, the ermE*p promoter was inserted upstream of *orf4* using plasmid pOJ260pNRPS (Table 1). The genotype of the resultant strain WT-pNRPS was confirmed by PCR: using oligonucleotides pNRPSDRV/M13FW and pNRPSIFW/M13RV (Table S1), a 2772 bp fragment and a 2686 bp fragment, were amplified from the recombinant strain but not from the WT strain (Figure S8). Additionally, *crxR1* was overexpressed in this strain, generating the strain WT-pNRPSeLuxR24. Comparisons of the metabolite profiles of these two recombinant strains and the WT-eLuxR24 did not reveal any additional peaks. All these results indicated that the production of the CRX compounds only required the “AHBA region” (now named *crx* BGC; see below), while the “NRPS region” was not involved in production of those compounds.

### 3.4. Determination of the Chemical Structure of Crexazones

**Figure 4:**
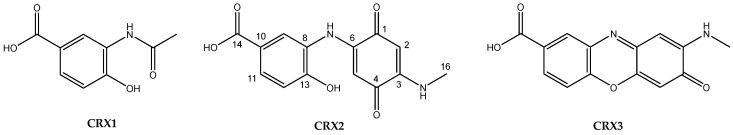
Chemical structures of crexazones.

The three major compounds produced by the WT-eLuxR24 strain were purified. They were named crexazone 1 (CRX1), CRX2, and CRX3 (Figure 4). The purification procedure afforded 5.4 mg of CRX1, 0.4 mg of CRX2, and 0.5 mg of CRX3. To assess the novelty of the compounds, dereplication by LC-DAD-HRMS analysis in combination with our in-house library [41] was performed first. In this analysis, CRX1, with the molecular formula C_9_H_9_NO_4_, was unambiguously identified as 3-acetamido-4-hydroxybenzoic acid (Figure S15), a derivative of 3,4-AHBA, which is a shunt product in the biosynthetic pathways of compounds such as cremeomycin [44], grixazone [55], and ahbamycins [56]. Likewise, CRX3, with the molecular formula C_14_H_10_N_2_O_4_, was identified as texazone (Figure S16), an aminophenoxazinone-type compound, similar to other compounds in this family such as maroxazinone [57] or APOC (2-aminophenoxazin-3-one-8-carboxylate) [58]. Texazone was discovered in *Actinomyces* strain WRAT-210, with its structure confirmed by chemical synthesis [59]. On the other hand, the LC-DAD-HRMS signature features of CRX2 were not found in our in-house dereplication library, and its molecular formula, C_14_H_12_N_2_O_5_, is not described in the Dictionary of Natural Products (DNP) [43], thus suggesting its putative novelty. Structural elucidation of CRX2 was consequently pursued.

A molecular formula of C_14_H_12_N_2_O_5_ was assigned to crexazone CRX2 based on ESI(+)-TOF data ([M + H]^+^ *m*/*z* 289.0821, calcd. 289.0819; Δ 0.69 ppm) (Figure S17). The joint analysis of ^1^H and HSQC NMR spectra acquired in DMSO-*d_6_* revealed the presence of three aromatic *sp2* hydrogens (H-9, H-11, and H-12), two olefinic *sp2* hydrogens appearing as singlets (H-2, H-5), and one methyl group appearing as a doublet (H-16), as well as at least two exchangeable protons (H-7, H-15) as a singlet and a quadruplet at δ_H_ 8.83 and 7.95, respectively (Table 2).

**Figure 5:**
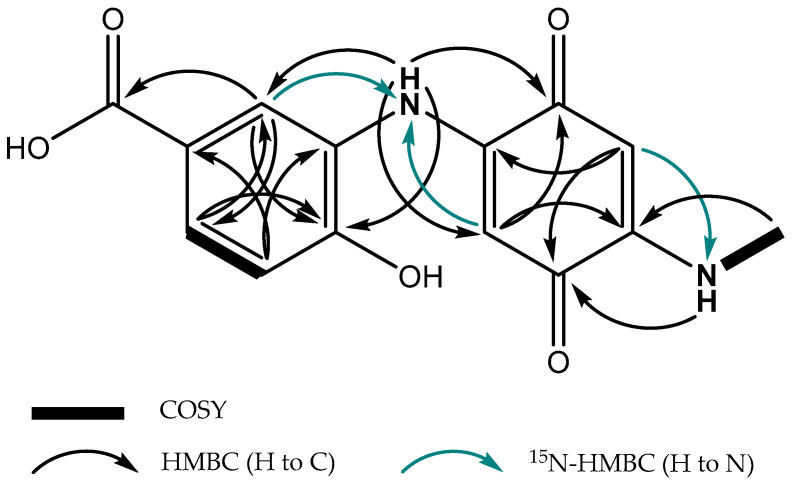
Key 2D NMR correlations observed in the structure of CRX2.

The spin system comprising H-9/H-11/H-12 in the COSY spectrum and their corresponding correlations in the HMBC spectrum allowed us to readily identify a 3,4-AHBA subunit in the structure of CRX2 (Figure 5). In turn, the characteristic ^1^H and ^13^C chemical shifts of olefinic signals 2 and 5 (δ_c_ 92.2, δ_H_ 5.31 ppm/δ_c_ 95.5, δ_H_ 5.50 ppm), their appearance as singlets, and their HMBC correlations with quinone-type carbonyls C-1 (δ_c_ 177.1) and C-4 (δ_c_ 179.4) strongly supported the presence of a *p*-quinone moiety in CRX2. This moiety was further determined as an *N*-methyl, 2-aminoquinone substructure comprising NH (H-15) and the methyl group H-16, based on COSY and HMBC correlations (Figure 5). In addition, the HMBC correlation from H-2 to C-6 revealed the substitution at this latter position. Finally, the *N*-linkage between C-6 and C-8 was established based on key HMBC correlations from NH (H-8) to C-1, C-5, C-9, and C-13, as well as ^15^N-HMBC correlations from both H-5 and H-9 to N-7 (Figure 5).

### 3.5. Generation of Mutants in Genes Related to N-N Bond Formation

**Figure 6:**
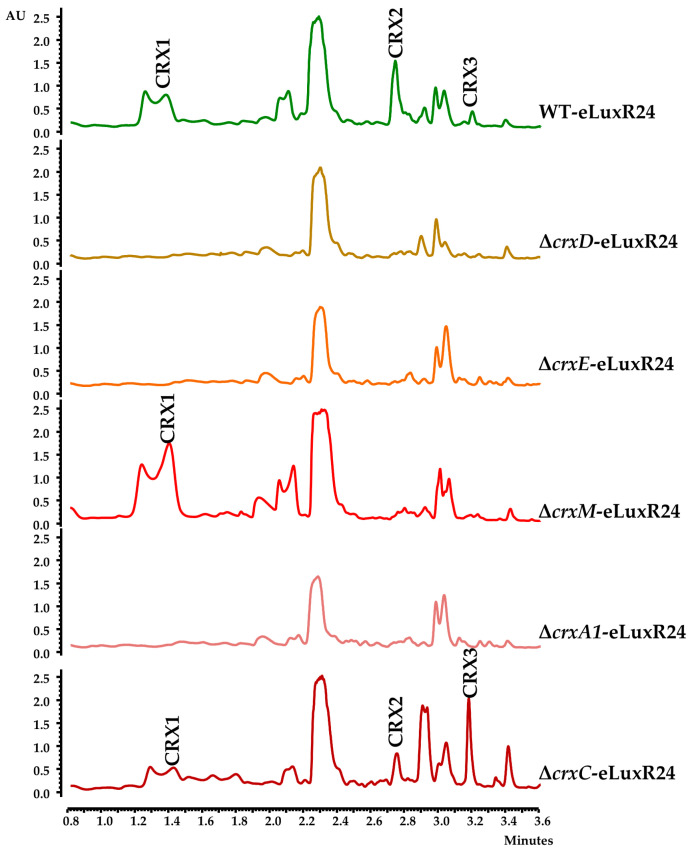
Production of crexazones by mutants in *crx* genes. UPLC Max Plot chromatograms of ethyl acetate-formic acid extracts of R5A cultures of WT-eLuxR24 in comparison to mutants in diazo-related genes (Δ*crxD*-eLuxR24, Δ*crxE*-eLuxR24, and Δ*crxM*-eLuxR24) and other *crx* genes (Δ*crxA1*-eLuxR24 and Δ*crxC*-eLuxR24). WT-eLuxR24, wild type overexpressing *orf24* (*crxR1*); Δ*crxD*-eLuxR24, Δ*crxD* mutant overexpressing *orf24* (*crxR1*); Δ*crxE*-eLuxR24, Δ*crxE* mutant overexpressing *orf24* (*crxR1*); Δ*crxM*-eLuxR24, Δ*crxM* mutant overexpressing *orf24* (*crxR1*); Δ*crxA1*-eLuxR24, Δ*crxA1*mutant overexpressing *orf24* (*crxR1*); Δ*crxC*-eLuxR24, Δ*crxC* mutant overexpressing *orf24* (*crxR1*). Peaks corresponding to different crexazones are indicated as CRX1, CRX2, and CRX3.

As mentioned above, CrxD (Orf16), CrxE (Orf17), and CrxM (Orf22) show similarity to CreD, CreE, and CreM from the cremeomycin BGC, which are involved in the formation of the diazo moiety in cremeomycin [15,16]. To assess their potential role in the biosynthesis of CRXs, their coding genes were individually deleted and replaced by the apramycin resistance cassette: (i) Mutant Δ*crxD* was constructed using plasmid pHZ1358Δ16 (Table 1) and was genetically confirmed by PCR: using oligonucleotides d16IFW/ApraI and d16DRV/ApraRV, a 2655 bp fragment A and a 2512 bp fragment B were amplified from the mutant but not from the WT strain, respectively (Figure S9). (ii) Mutant Δ*crxE* was generated using pHZ1358Δ17 (Table 1), and it was confirmed by PCR: using oligonucleotides d17IFW/ApraI and d17DRV/ApraFW, a 2595 bp fragment A and a 3677 bp fragment B were amplified from the mutant but not from the WT strain, respectively (Figure S10). (iii) Mutant Δ*crxM* was constructed with plasmid pHZ1358Δ22 (Table 1). Its genotype was confirmed by PCR: using oligonucleotides d22IFW/ApraI and d22DRV/ApraD, a 2907 bp fragment A and a 3035 bp fragment B were amplified from the mutant but not from the WT strain, respectively (Figure S11). After expressing the *crxR1* regulator into these mutants, culture extracts from the resultant recombinant strains (Δ*crxD*-eLuxR24, Δ*crxE*-eLuxR24, and Δ*crxM*-eLuxR24) were analyzed for CRX production (Figure 6).

UPLC analyses of organic extracts showed that CRXs production was abolished in Δ*crxD*-eLuxR24 and Δ*crxE*-eLuxR24, indicating the essential roles of CrxD and CrxE in CRXs biosynthesis. Deletion of *crxM* in Δ*crxM*-eLuxR24 resulted in blocking CRX2 and CRX3 production, which contain two linked moieties related to 3,4-AHBA, while CRX1 was still produced. These findings suggest that CrxM, a homolog of CreM ligase involved in incorporating the diazo group in cremeomycin [16], plays an essential role in the biosynthesis of CRX2 and CRX3.

Additionally, mutants were generated in *crxA1* and *crxC*, which encode proteins with domains that are also present in Spb39 and Spb40 enzymes involved in the biosynthesis of a hydrazine group in the S56-p1 antibiotic [48] (Figure S1). *crxA1* encodes an FAD-dependent oxidoreductase that, similarly to Spb39, contains DadA and DAO domains. CrxC contains cupin_1 and cupin_2 domains, which are also found in Spb40. Mutant Δ*crxA1*, generated using plasmid pHZ1358Δ11, was confirmed by PCR using oligonucleotides d11IFW/ApraI and d11DRV/ApraD, which amplify a 2337 bp fragment A and a 2627 bp fragment B from the mutant but not from the WT strain, respectively (Figure S12). Mutant Δ*crxC*, constructed using pHZ1358Δ14, was confirmed by PCR using oligonucleotides d14IFW/ApraI and d14DRV/ApraD, which amplify a 3662 bp fragment A and a 2181 bp fragment B from the mutant but not from the WT strain (Figure S13). After *crxR1* was expressed in these two mutants, the resultant strains were cultivated, and their metabolite profiles were analyzed by UPLC. It was observed that production of CRXs was not affected in Δ*crxC*-eLuxR24, while it was abolished in Δ*crxA1*-eLuxR1 (Figure 6). This indicates that CrxA1 but not CrxC, is essential for CRXs production in the tested conditions. Complementation of the Δ*crxA1* mutant was achieved (Figure S14) using plasmid pSETEcHc11LuxR24 (Table 1). This conclusively demonstrates its involvement in crexazone production.

## 4. Discussion

Genome mining using antiSMASH has proven useful for discovering new BGCs that encode novel compounds that are potentially active. However, there are challenges in detecting certain types of clusters, such as those related to the biosynthesis of aminohydroxybenzoates [19,56]. In this study, we have used BLAST analysis to search for genes involved in the formation of *N-N* bonds in genomes from the *Streptomyces* CS strain collection. This has led to us identifying two genes related to diazo group formation (*crxD* and *crxE*) in the CS057 strain. These genes were located at the NRPS BGC 28 identified by antiSMASH, within a region that also contained genes for the biosynthesis of 3,4-AHBA (*crxH* and *crxI*). The whole DNA region, highly conserved in several *Streptomyces* strains, was silent or poorly expressed under the tested culture conditions. Overexpression of the LuxR-like regulator *crxR1*, combined with deletion of selected genes, allowed the activation of the *crx* BGC, the identification of its encoded CRX compounds, and the discard of the involvement of the NRPS coding gene in their biosynthesis. The three major CRX compounds were purified and chemically characterized. They derive from 3,4-AHBA and correspond to 3-acetamido-4-hydroxybenzoic acid (CRX1), also known as AHB119 [56] and 3,4-AcAHBA [55], which contains one unit of 3,4-AHBA; the phenoxazinone CRX3 (also known as texazone [59]), with two linked 3,4-AHBA moieties; and the novel quinone-containing compound CRX2, both containing two moieties derived from 3,4-AHBA.

Unexpectedly, none of the CRX compounds have a diazo group, despite the fact that the *crx* BGC contains two genes (*crxD* and *crxE*), encoding similar proteins to those involved in nitrous acid biosynthesis for the formation of diazo groups [16,17,18,19,20,21,44]. Both CrxE and CrxD contain the same domains as their homologs. Moreover, CrxD conserves all the amino acid residues that have been shown to be essential for CreD activity [60]. In addition to cremeomycin BGC, homologs to CrxD and CrxE are found in other BGCs, such as alazopeptin (AzpD and AzpE) [18], tasikamydes (Aha1 and Aha2) [19], avenalumic acid (AvaD and AvaE) [20], spinamycin (SpiED) [17], or triacsins (Tri16 and Tri21) [21], among others. In these BGCs, CreD and CreE homologs are involved in the formation of nitrous acid, which is used by a CreM-like ligase to synthesize a diazo group in these compounds [16,17,20,21]. However, deletion of *crxD* or *crxE* in CS057 abolished the production of CRX compounds, even CRX1, which is 3,4-AcAHBA, indicating that these two genes play an essential role in the biosynthesis of CRXs. In this regard, it might be possible that the formation of the diazo group could take place, but CRX compounds containing these functional groups are not detected. Although experiments were conducted under dark conditions to avoid compound degradation, it cannot rule out the degradation of these putative compounds because of their photosensitivity. This happens with cremeomycin, which is degraded to 2,4-HMBA (2-hydroxy-4-methoxybenzoic acid) within hours to days, even in the absence of light [44]. Alternatively, it is possible that the formation of a diazo group could be a biosynthetic cryptic step in the overall pathway, required for further modification steps in the biosynthesis of CRXs. The existence of biosynthetic cryptic steps has been described in different types of biosynthetic pathways [61,62]. The formation of diazo groups has also been described as a cryptic step in the avenalumic acid pathway, where it is subsequently replaced by a hydride group [20]; in tasikamides biosynthesis, where the formation of the diazo group leads to the non-enzymatic generation of a hydrazone group [19]; or in the biosynthesis of three novel desferrioxamine derivatives containing five-membered heterocyclic ring structures [63]. Deletion of *crxA1* also stops production of any CRX compound without accumulating any structurally related compound, which makes it difficult to assign it a role. The lack of production of CRX1 by all these three mutants could indicate the need for a minimal concentration of biosynthetic intermediates to act as inducers. This also happens during chromomycin biosynthesis, where blocking the glycosylation of the aglycone prevents the accumulation of any aglycone intermediate [64].

**Figure 7:**
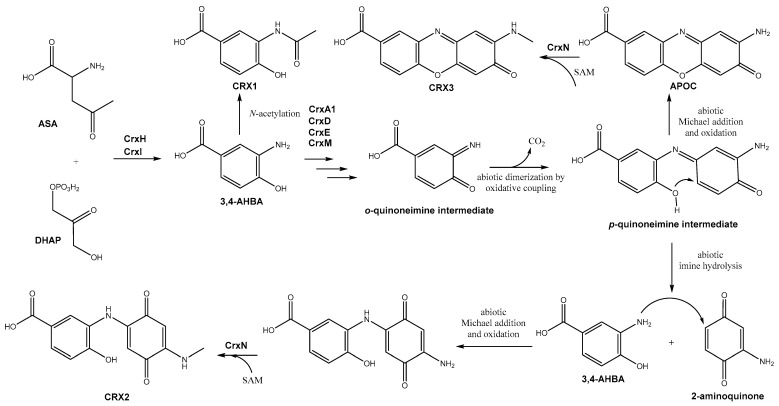
Proposed biosynthetic pathway for CRX compounds.

Taking into consideration all previously stated observations, protein homology analyses, and proposed biosynthetic pathways for other 3,4-AHBA-derived compounds, a biosynthetic pathway is proposed for CRXs (Figure 7). The first step would consist of the biosynthesis of 3,4-AHBA from aspartate semialdehyde (ASA) and dihydroxyacetone-P (DHAP) catalyzed by CrxH and CrxI, similarly to that reported in the biosynthesis of 3,4-AHBA in the grixazone biosynthetic pathway [65]. CRX1 would be a shunt product, which has also been identified in the biosynthetic pathway of ahbamycins and grixazone [55,56]. The formation of this compound only requires the N-acetylation of 3,4-AHBA. As found in the ahbamycin and grixazone biosynthetic gene clusters, the *crx* BGC does not contain any acetyltransferase-coding genes. Therefore, this gene should be located elsewhere in the genome. CRX3 is identical to the phenoxazinone texazone [59], an N-methylated derivative of APOC [58]. APOC has been identified as a shunt product in the biosynthetic pathway for grixazone [58,65]. In that pathway, the formation of APOC is preceded by the oxidation of 3,4-AHBA to its *o*-quinoneimine, which dimerizes to generate APOC with a concomitant loss of carboxyl group. The phenoxazinone synthase GriF (and the chaperone GriE) catalyzes the formation of the mentioned *o*-quinoneimine, and the subsequent coupling step to render the phenoxazinone ring system is supposed to proceed non-enzymatically [65]. The biosynthesis of APOC in the CRX pathway could follow the same route. However, the *crx* BGC lacks any similar gene to *griF*-encoding a phenoxazinone synthase. Nevertheless, there are two BGCs in CS057 that contain genes coding for similar proteins to GriF and GriE: BGC 4 (WP_087763898.1 and WP_087763899.1) and BGC 21 (WP_087765103.1 and WP_087765102.1). These proteins could play the same role as GriF and GriE for *o*-quinoneimine formation. Alternatively, the biosynthesis of CRX3 could follow another pathway. Thus, the formation of the *o*-quinoneimine from 3,4-AHBA could be carried out by CrxA1, which contains a DAO domain also present in enzymes that catalyzes the oxidation of C-N bonds to yield imine groups. In accordance with this, *crxA1* plays an essential role in CRX biosynthesis since its deletion completely abolished the production of all CRX compounds. Moreover, three other proteins (CrxD, CrxE, and CrxM) should be involved in the biosynthesis of CRX3 (and CRX2), given that the deletion of their coding genes block the biosynthesis of CRX3 (and CRX2). Since mutants in *crxA1*, *crxD,* or *crxE* do not produce any CRX compound, while the mutant in *crxM* still produces CRX1, CrxM should act after the others. CRX2 is a novel 3,4-AHBA-derived compound that contains a 3,4-AHBA unit that is *N*-alkylated by 2-aminoquinone. This *p*-quinone, ultimately derived from 3,4-AHBA as later proposed, would be *N*-alkylated by 3,4-AHBA itself through abiotic Michael addition followed by reoxidation to *p*-quinone via a non-enzymatic process. We hypothesize that the 2-aminoquinone, which acts as the Michael acceptor in the route towards CRX2, could be derived from the hydrolysis of the *p*-quinoneimine intermediate [66,67] in the abiotic formation of the phenoxazinone ring system of APOC (Figure 7). The last step in CRX2 and CRX3 biosynthesis would be an N-methylation step. This could be carried out by the methyltransferase CrxN. Overall, these results suggest the involvement of diazo-forming enzymes in an unexpected biosynthetic step: the oxidation of *o*-aminophenols to *o*-quinoneimines. Further studies would be required to clarify these biosynthetic steps and determine the role of the different *crx* gene products.

## 5. Conclusions

By using diazo-synthesis related genes to in silico screening genomes from twelve *Streptomyces* strains belonging to the “CS” collection, we have uncovered the new and silent *crx* BGC in *Streptomyces* sp. CS057. This cluster was activated overexpressing the LuxR-like regulatory gene *crxR1*, which has led to the identification of its encoded compounds named crexazones. These compounds derive from 3,4-AHBA but lack a diazo group. Generation of mutants in specific genes experimentally proved the involvement of *crx* BGC in the biosynthesis of those compounds. Mutations in *crx* diazo-synthesis related genes suggest their essential and unexpected role in crexazones biosynthesis. Further experiments would be required to clarify the role of these *crx* gene products in crexazones biosynthesis.

## Figures and Tables

**Table 1:** Strains generated in this study.

**Mutant Strain**	**Plasmid**	**Deleted Genes**
ΔAHBA	pHZ1358ΔAHBA	*orf14-23*
ΔNRPS	pHZ1358ΔNRPS	*orf4*
Δ*crxA1*	pHZ1358Δ11	*crxA1*
Δ*crxC*	pHZ1358Δ14	*crxC*
Δ*crxD*	pHZ1358Δ16	*crxD*
Δ*crxE*	pHZ1358Δ17	*crxE*
Δ*crxM*	pHZ1358Δ22	*crxM*
ΔTetR	pHZ1358ΔTetR	*crxR2*
**Recombinant Strain**	**Plasmid**	**Expressed Genes**
WT-eLuxR24	pSETEcHLuxR24	*crxR1*
WT-eLuxRA	pSETEcHLuxRA	*orfA*
Wt-eLuxR24A	pSETEcHLuxR24A	*orfA*, *crxR1*
WT-kLuxR24	pSETxkLuxR24	*crxR1*
WT-pLuxR24	pOJ260pLuxR24	*crxR1*
WT-kTetR	pSETxkTetR	*crxR2*
WT-pNRPS	pOJ260pNRPS	*orf4*
WT-pNRPS-eLuxR24	pOJ260pNRPS	*orf4*
	pSETEcHLuxR24	*crxR1*
ΔAHBA-eLuxR24	pSETEcHLuxR24	*crxR1*
ΔNRPS-eLuxR24	pSETEcHLuxR24	*crxR1*
Δ*crxA1*-eLuxR24	pSETEcHLuxR24	*crxR1*
Δ*crxC*-eLuxR24	pSETEcHLuxR24	*crxR1*
Δ*crxD*-eLuxR24	pSETEcHLuxR24	*crxR1*
Δ*crxE*-eLuxR24	pSETEcHLuxR24	*crxR1*
Δ*crxM*-eLuxR24	pSETEcHLuxR24	*crxR1*
Δ*crxA1c*	pSETEcHc11LuxR24	*crxR1*, *crxA1*

**Table 2:** NMR Data (500 MHz, DMSO-*d_6_*, at 24 °C) for crexazone 2 (CRX2).

Position	δ_C_	δ_H_ (mult, *J* in Hz)
1	177.1, C	
2	92.2, CH	5.31, s
3	151.5, C	
4	179.4, C	
5	95.5, CH	5.50, s
6	147.1, C	
7		8.83, s
8	125.0, C	
9	124.0, CH	7.78, d (1.9)
10	121.8, C	
11	128.3, CH	7.69, dd (8.4, 1.9)
12	115.7, CH	7.03, d (8.4)
13	154.3, C	
14	166.7, C	
15		7.95, q (5.3)
16	29.0, CH_3_	2.78, d (5.3)

## Data Availability

The data presented in this study are available in the article and in the Supplementary Materials.

## Supplementary Materials

The following supporting information can be downloaded at: https://www.mdpi.com/article/10.3390/biom14091084/s1. Table S1: Oligonucleotides used for PCR amplification; Table S2: Similar gene products to CreD and CreE in the CS strain collection; Table S3: Predicted functions of genes in the *Streptomyces* sp. CS057 *crx* BGC; Figure S1: Types of *N-N* bonds and proteins involved in their formation; Figure S2: Generation of the CS057ΔAHBA mutant strain; Figure S3: Generation of the CS057ΔNRPS mutant strain; Figure S4: Comparison of metabolite profiles of ΔAHBA and ΔNRPS mutants and the wild type strain CS057; Figure S5: Generation of ΔTetR mutant; Figure S6: Comparison of metabolite profiles of wild type strain CS057 with ΔTetR and WT-kTetR; Figure S7: Generation of the WT-pLuxR24 recombinant strain; Figure S8: Generation of the WT-pNRPS recombinant strain; Figure S9: Generation of the Δ*crxD* mutant strain; Figure S10: Generation of the Δ*crxE* mutant strain; Figure S11: Generation of the Δ*crxM* mutant strain; Figure S12: Generation of the Δ*crxA1* mutant strain; Figure S13: Complementation of ΔcrxA1 mutant; Figure S14: Generation of the Δ*crxC* mutant strain; Figure S15: Identification of CRX1 by dereplication; Figure S16: Identification of CRX1 by dereplication; Figure S17: UV (DAD) spectrum and HR-ESI(+)-TOF MS spectrum of CRX2; Figure S18: ^1^H NMR spectrum of CRX2; Figure S19: ^13^C NMR spectrum of CRX2; Figure S20: COSY spectrum of CRX2; Figure S21: HSQC spectrum of CRX2; Figure S22: HMBC spectrum of CRX2; Figure S23: ^15^N-HMBC spectrum of CRX2. References [11,15,45,47,48] are cited in Supplementary Materials.
