# Supplementary material for: Genome Mining for Diazo-Synthesis-Related Genes in Streptomyces sp. CS057 Unveiled the Cryptic Biosynthetic Gene Cluster crx for the Novel 3,4-AHBA-Derived Compound Crexazone 2
Source: Biomolecules. 2024 Aug 29;14(9):1084. doi: 10.3390/biom14091084 (PMC11429834; doi:10.3390/biom14091084)
Supplement: Supplementary file 1 [file biomolecules-14-01084-s001.zip › biomolecules-3156015-supplementary.pdf]

Supplementary materials

Genome mining for diazo synthesis related genes in *Streptomyces* sp. CS057 unveiled the cryptic biosynthetic gene cluster *crx* for the novel 3,4-AHBA derived compound crexazone 2

Laura Prado-Alonso [1,2], Suhui Ye [1,2], Ignacio Pérez-Victoria [3], Ignacio Montero [1,2], Pedro Riesco [1,2], Francisco Javier Ortiz-López[3], Jesús Martín[3], Carlos Olano [1,2], Fernando Reyes [3], and Carmen Méndez [1,2*]

[1] **Departamento de Biología Funcional e Instituto Universitario de Oncología del Principado de Asturias (IUOPA), Universidad de Oviedo, 33006 Oviedo, Spain.**

[2] **Instituto de Investigación Sanitaria de Asturias (ISPA), 33011 Oviedo, Spain.**

[3] **Fundación MEDINA, Centro de Excelencia en Investigación de Medicamentos Innovadores en Andalucía, Armilla, 18016 Granda, Spain.**

*** Correspondence: cmendezf@uniovi.es**

Table S1. Oligonucleotides used for PCR amplification.

Oligonucleotide	Sequence 5′-3′
Mut1A_BglII	AAAAGATCTCATGCCCAGGAGACCATC
Mut1B_EcoRI	AAAGAATTCCCGACCACCTCCGTGTAG
Mut2ABglII	AAAAGATCTCGAGGACTTCGGCATGAT
Mut2BEcoRV	AAAGAATTCCGCGTCTGTGTGATGACC
AdNRPS FW	CCGCCGAAAGTTCCTCGAAGATCTGTCCTGGCTGTTTGG
AdNRPS RV	GGCATCAGTTACCGTGAGCCGGGTCAGACATGCACTC
BdNRPS FW	CTTGACATTGGGGAATTGACGGACCCGTACGAGATCGAC
BdNRPS RV	CGGGGACCTGCAGGTCGACTCGTCAAGCAGACCATTCC
ApraGibs FW	GCTCACGGTAACTGATGCC
ApraGibs RV	CGTCAATTCCCCAATGTCAAG-3
d11AFW	CCGCCGAAAGTTCCTCGAAGCGCGTACAGCTTCAACAG
d11ARV	GGCATCAGTTACCGTGAGCGTGTTCACCCATGGTTCAG
d11BFW	CTTGACATTGGGGAATTGACGTGACGGCCGCTCCCAC
d11BRV	CGGGGACCTGCAGGTCGACTGGGCGCCAAGCACAAATTC
d14AFW	CCGCCGAAAGTTCCTCGAAGTCCCAGCGTGTCTTG
d14ARV	GGCATCAGTTACCGTGAGCCGCCTTCACCGTGTACTTG
d14BFW	CTTGACATTGGGGAATTGACGCGAGGACTTCGGCATGATC
d14BRV	CGGGGACCTGCAGGTCGACTTTCTGTGCCCACGAACC
d16AFW	CCGCCGAAAGTTCCTCGAAGCGGGTAGTCCGATTGTTC
d16ARV	GGCATCAGTTACCGTGAGCGGTCATGAGGCTCCTTGG
d16BFW	CTTGACATTGGGGAATTGACGCACGCGGCCCGTTGAC
d16BRV	CGGGGACCTGCAGGTCGACTCGCCTTCACCGTGTACTTG
d17AFW	CCGCCGAAAGTTCCTCGAAGTCGCTGACCAGGTACTTG
d17ARV	GGCATCAGTTACCGTGAGCGCCGACCACACAGATTC
d17BFW	CTTGACATTGGGGAATTGACGCCGACGGCCCAAGGAGC
d17BRV	CGGGGACCTGCAGGTCGACTAACTACGGCCTCTGGAAC
d22AFW	CCGCCGAAAGTTCCTCGAAGTCGACTGGCTCATCC
d22ARV	GGCATCAGTTACCGTGAGCCATGCGTTCCTTCCTG
d22BFW	CTTGACATTGGGGAATTGACGACGGTCCTGCTGAAG
d22BRV	CGGGGACCTGCAGGTCGACTCGACCGTGAAGAAGG
dTetRAFW	CCGCCGAAAGTTCCTCGAAGCGCGAGAACATCGTC
dTetRARV	GGCATCAGTTACCGTGAGCCGGTCGGCCTCTTTC
dTetRBFW	CTTGACATTGGGGAATTGACGGCCGTGACCCTCTGG
dTetRBRV	CGGGGACCTGCAGGTCGACTTACGGCACCAAGCTC
cORF11Lux24c5Lux24EcoRVFW	GCTGAGCCGATGGCCTGATTTTCTGTGCCCACGAACC
cORF11Lux24c5pSETHEcoRVRV	GACCATGATTACGAATTCGATGTCACGGCGCGACCAGAG
eLuxR24XbaIFW	GCTCTAGAATGGGCAACGATGTG
eLuxR24EcoRVRV	CGAGATATCAGGCCATCGGCTCAG
eLuxRAEcoRVFW	GCAGATATCAGACGGACAGAGGGAGAG
eLuxRAEcoRVRV	TAAGATATCGTTCCGGGAGTCAGTTGC
orfApSETHGibsFWXbaI	GTAGGATCCAGACCTGCAGTCAGACGGACAGAGGGAGAG
orfApSETHGibsRVcorf24	CTGAATTACTTCCGGCCTCCTGGTTCCGGGAGTCAGTTGC
orf24FW	CAGGAGGCCGGAAGTAATTCAG
orf24gibsRVEcoRV	CTATGACCATGATTACGAATTCGATAGGCCATCGGCTCAG
FW24c3pSETxkBamHI	GTTACTAGGGTCGACTCTAGAGGAGGCCGGAAGTAATTCAG
RV24c5pSETxkEcoRV	CGTGGCGGGTTGGGCCGATAGGCCATCGGCTCAG
kTetRcpSETxkBamHIFW	ATCGGATCCTCCGAGGAGGCAGAC
kTetRcpSETxkEcoRVRV	TACCATATGCCGGCCCTGATTTCC
pNRPSXbaIFW	GAGTCTAGAGGGCGGCGATGTATG

pNRPSEcoRIRV	CAAGAATTCCCGGTCGAGGTAGTG
CS057MutCA	ACCGGAAAGAGCGAACT
ApraCRV	TCATTCTGTGGGCCGTAC
CS057MutCB	AGGTCGACATCCCGTTC
ApraCFW	AAGTGCTTGACATTGGGG
dNRPSIFW	CGTCGTAGTACGTGTC
ApraI	GAAGCTGACCGATGAG
dNRPSDRV	GTCAAGCACCCGAAG
ApraD	ACATTGTGGCGACAGC
dTetRIFW	TCGGACAGCGATGTG
dTetRDRV	GGGTTCCAGGTCATGAG
M13FW	GTAAAACGACGGCCAGT
eLuxR24DRV	GCGTTCCTTCCTGGGAG
pNRPSDRV	CAGGTACTCCAGCACAC
pNRPSIFW	CGTTCGGCTCTTTCC
M13RV	CAGGAAACAGCTATGAC
d16IFW	GCTGCTGGCATATGTC
d16DRV	CCACGAGCTGTTCGTAG
d17IFW	GGCCTGGAAGATGTTG
ApraFW	TCAGCTTCTCAACCTTGG
d17DRV	ACCCGCTACTGGTTC
d22IFW	TCATGCCCAGGAGAC
d22DRV	TGCGCAAGAAGAACG
d11IFW	AGGTCGTCGGTGTAGAG
d11DRV	CGGGTGAAGAAGTCATC
d14IFW	GACCCGACCGGCCTGAC
d14DRV	ACGAATCACGCGAACG

Table S2. Similar gene products to CreD and CreE the in the CS strain collection.

		CreD (ALA99201.1)	CreE (ALA99202.1)
**CS014**	Accession	WP_109195827.1	WP_109195828.1
	% identical aminoacids	68,96	61,37
**CS057**	Accession	WP_087766625.1	WP_087766626.1
	% identical aminoacids	58,85	54,24
**CS065a**	Accession	WP_109167419.1	WP_109164827.1
	% identical aminoacids	65,81	60,37
**CS131**	Accession	WP_109180564.1	WP_109180563.1
	% identical aminoacids	68,29	61,76
**CS147**	Accession	WP_098898954.1	WP_109176115.1
	% identical aminoacids	68,51	61,62
**CS149**	Accession	WP_109195827.1	WP_109195828.1
	% identical aminoacids	68,96	61,37
**CS207**	Accession	WP_109204078.1	WP_109204002.1
	% identical aminoacids	56,04	61,04

Table S3. Predicted functions of genes in the *Streptomyces* sp. CS057 *crx* BGC.

Streptomyces sp. CS057				*Streptomyces* sp. MNU77		*Streptomyces* sp. TSRI0395		*Streptomyces sp. TSRI0261*	
orf	*Crx*	Accession	Product	% iden	Accession	% iden	Accession	% iden	Accession
A		WP_087766615.1	Helix-turn-helix domain-containing protein, LuxR family	36,62	WP_047179483.1	92,1	WP_073958791.1	93,01	OKJ12698.1
1		WP_178798375.1	acyl carrier protein	100	WP_178798375.1	96,3	WP_198958491.1	97,4	OKJ13302.1
2		WP_087767999.1	3-hydroxyacyl-ACP dehydratase	99,35	WP_047180651.1	96.48	WP_073959081.1	88,3	WP_073801542.1
3		WP_087766616.1	3-hydroxyacyl-ACP dehydratase	98,23	WP_047176462.1	89,38	WP_073958792.1	91,15	WP_069754598.1
4		WP_087766617.1	non-ribosomal peptide synthetase	99,09	WP_052658795.1	93,76	WP_073958793.1	95,31	-
5		WP_087766618.1	hypothetical protein	99,15	WP_178798377.1	95,92	WP_079185305.1	96.95	OKJ13303.1
6		WP_087768000.1	hppD (4-hydroxyphenylpiruvate dioxygenase)	98,52	WP_047180648.1	93,49	WP_073958794.1	95,56	OKJ13304.1
7		WP_087768001.1	aminotransferase class I/II-fold pyridoxal phosphate-dependent enzyme	98,03	WP_075347939.1	94,94	WP_073959083.1	94,56	OKJ13305.1
8		WP_087766619.1	xanthine dehydrogenase accessory protein XdhC	99,26	WP_047176461.1	96,27	WP_073958796.1	98,4	OKJ12701.1
9		WP_087766620.1	xanthine dehydrogenase molybdopterin binding subunit	99,13	WP_047176460.1	95,9	WP_073958797.1	96,4	OKJ12702.1
10		WP_087766621.1	FAD binding domain-containing protein	98,77	WP_047176459.1	96,51	WP_073958798.1	96,3	OKJ12703.1
11	*crxA1*	WP_047176458.1	FAD-dependent oxidoreductase	100	WP_047176458.1	99.73	WP_050361360.1	100	WP_047176458
12	*crxA2*	WP_087766622.1	NAD(P)H-binding protein	98,69	WP_047176457.1	96,73	WP_073958801.1	95,42	OKJ12708.1
13	*crxB*	WP_087766623.1	DsbA family protein	98,68	WP_047176456.1	96,92	WP_057662511.1	97,8	WP_073801557.1
14	*crxC*	WP_052658793.1	cupin domain-containing protein	100	WP_052658793.1	99,33	WP_050361358.1	99,33	OKJ12710.1
15	*crxA3*	WP_179233816.1	FAD-binding protein	98,89	WP_079191042.1	98,7	WP_098892422.1	98,89	OKJ12711.1
16	*crxD*	WP_087766625.1	pcaB, 3-carboxy-cis,cis-muconate cycloisomerase	99,15	WP_047176453.1	97,68	WP_073958802.1	96,87	OKJ12712.1
17	*crxE*	WP_087766626.1	FAD/NAD(P)-binding protein	99,1	WP_063780445.1	97,01	WP_073959084.1	97,6	OKJ13307.1
18	*crxK*	WP_079191147.1	aspartate kinase	100	WP_079191147.1	99,52	WP_079044364.1	99,5	OKJ12713.1
19	*crxI*	WP_087766627.1	2-amino-3,7-dideoxy-D-threo-hept-6-ulosonate synthase	99,63	WP_047176451.1	98,46	WP_073958803.1	98,07	OKJ12714
20	*crxH*	WP_047176450.1	3-dehydroquinate synthase II	100	WP_047176450.1	99,19	WP_007449449.1	98,92	OKJ12715.1
21	*crxN*	WP_087766628.1	methyltransferase	99,71	WP_047176449.1	99,71	WP_050361353.1	99,71	OKJ12716.1
22	*crxM*	WP_087766629.1	acyl-CoA ligase	99,48	WP_052658792.1	99,48	WP_050361352.1	99,11	OKJ13308.1
23	*crxF*	WP_007449445.1	VOC family protein	100	WP_007449445.1	100	WP_007449445.1	99,17	OKJ12717.1
24	*crxR1*	WP_179233817.1	LuxR family transcriptional regulator	99,28	WP_075347773.1	97.25	WP_073958804.1	98,21	OKJ12718
25	*crxT*	WP_047176447.1	multidrug efflux SMR transporter	100	WP_047176447.1	99,04	WP_032791178.1	98,08	OKJ12719.1
26	*crxP*	WP_032791177.1	3-oxoacyl-ACP reductase FabG	100	WP_032791177.1	100	WP_032791177.1	100	OKJ12720.1
27	*crxR2*	WP_056706759.1	TetR/AcrR family transcriptional regulator	100	WP_047176446.1	100	WP_047176446.1	100	OKJ12721.1
28		WP_087766631.1	hypothetical protein	99,25	WP_047176445.1	78,49	WP_073959085.1		
29		WP_087766632.1	hypothetical protein	97	WP_047176444.1	91,91	WP_073958805.1		
30		WP_087766633.1	hypothetical protein	93,87	WP_047176443.1	93,29	WP_073958806.1		

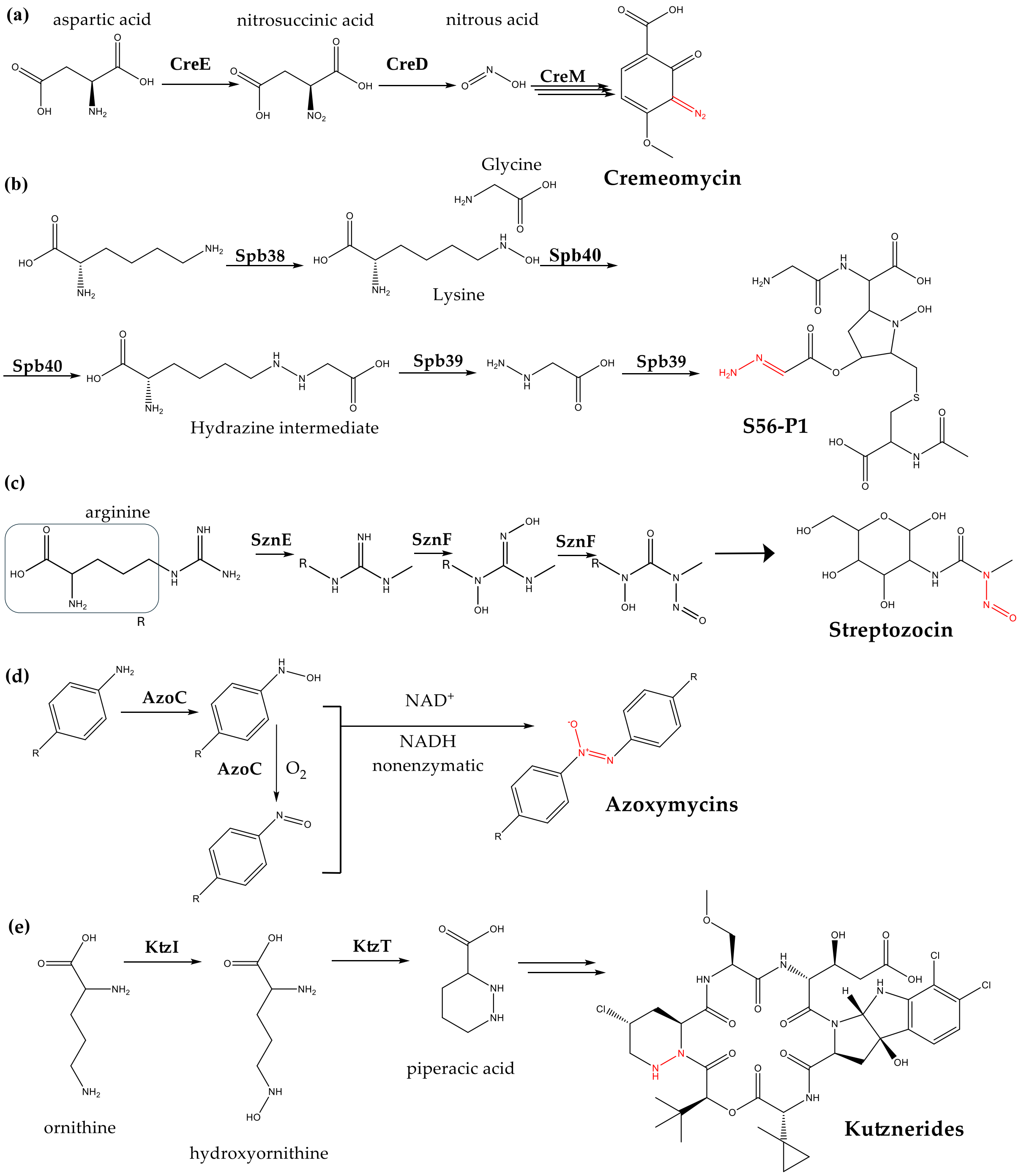

Figure S1. Types of *N-N* bonds and proteins involved in their formation: diazo group in cremeomycin [15]; hydrazine group in s56-p1 [48]; N-nitroso group in streptozocin [47]; azoxy group in azoxymycins [45]; piperazic acid in kutznerides [11].

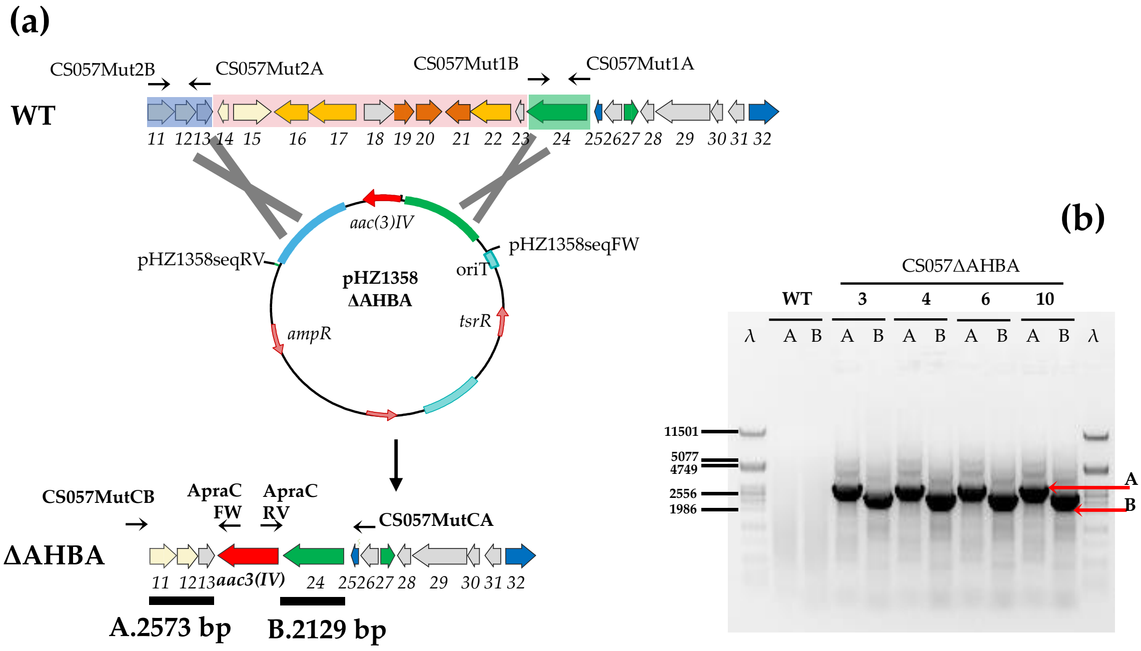

Figure S2. Generation of the CS057ΔAHBA mutant strain. (**a**) Scheme representing the construction of the CS057ΔAHBA mutant by gene replacement of *orf14-23* by the apramycin resistance gene using pHZ1358ΔAHBA. (**b**) PCR analysis of ΔAHBA using oligonucleotides CS057MutCA and ApraCRV (A) and oligonucleotides CS057MutCB and ApraCFW (B) WT, CS057 wild type strain; 3, 4, 6 and 10 correspond to four independent ΔAHBA mutants; λ, PstI-digested Lambda DNA; *aac3(IV)*, apramycin resistance gene; *ampR*, ampicillin resistance gene; *tsrR*, thiostrepton resistance gene.

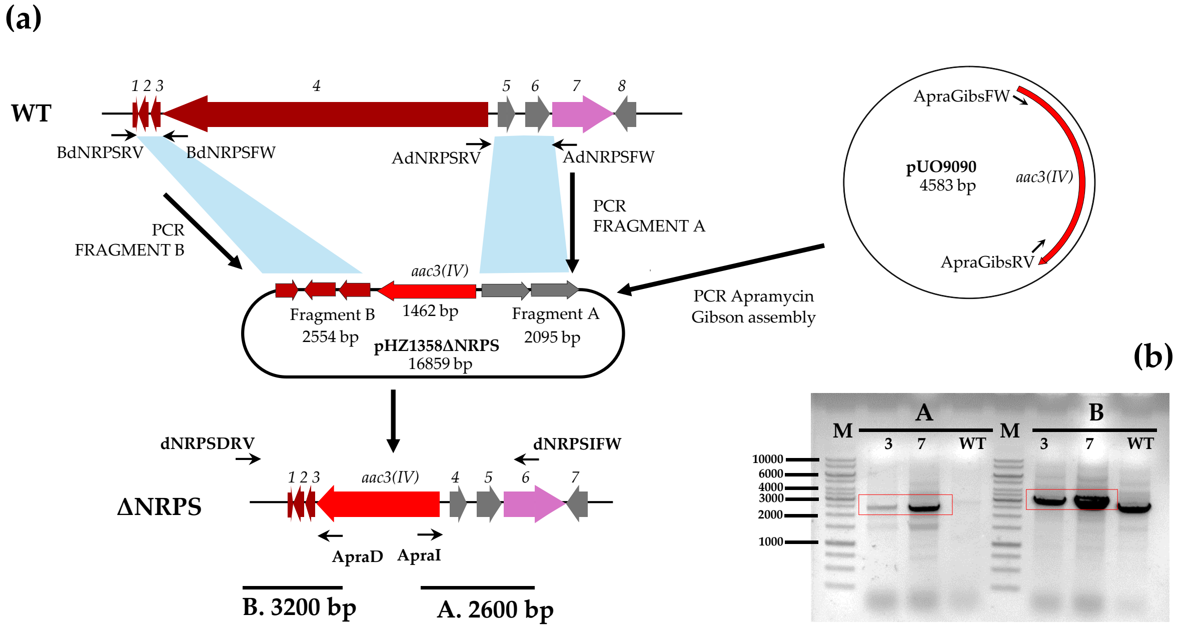

Figure S3. Generation of the CS057ΔNRPS mutant strain. (**a**) Scheme representing the construction of pHZ1358ΔNRPS by Gibson assembly, and the generation of the ΔNRPS mutant by gene replacement of *orf4* by the apramycin resistance gene. (**b**) PCR confirmation of ΔNRPS using oligonucleotides dNRPSIFW and ApraI (A) and oligonucleotides dNRPSDRV and ApraD (B). 3 and 7 correspond to two independent ΔNRPS mutants. WT, wild type strain; *aac3*(IV), apramycin resistance gene; M, DNA ladder (Generuler 1 kb).

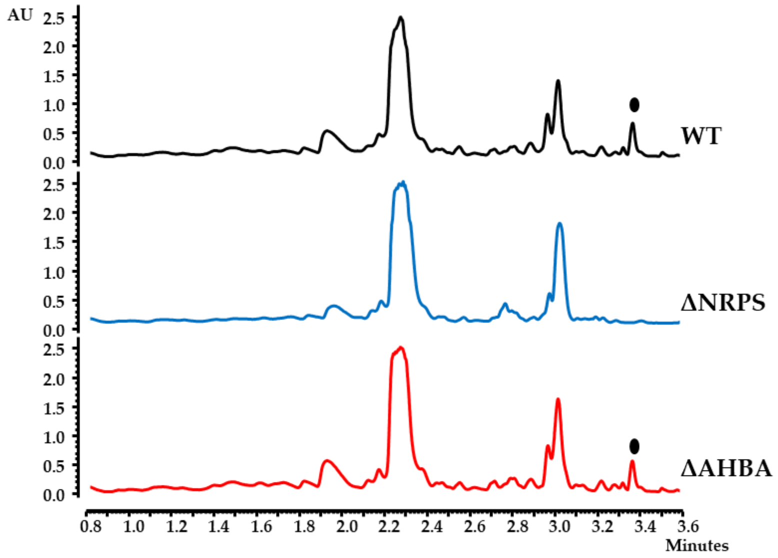

Figure S4. Comparison of metabolite profiles of ΔAHBA and ΔNRPS mutants and the wild type strain CS057. UPLC chromatograms (Max Plot) of ethyl acetate-1 % formic acid extracts of 5 days-old cultures in R5A medium. WT, wild type strain. Black point (●) indicates a differential compound unrelated with the Orf4 NRPS.

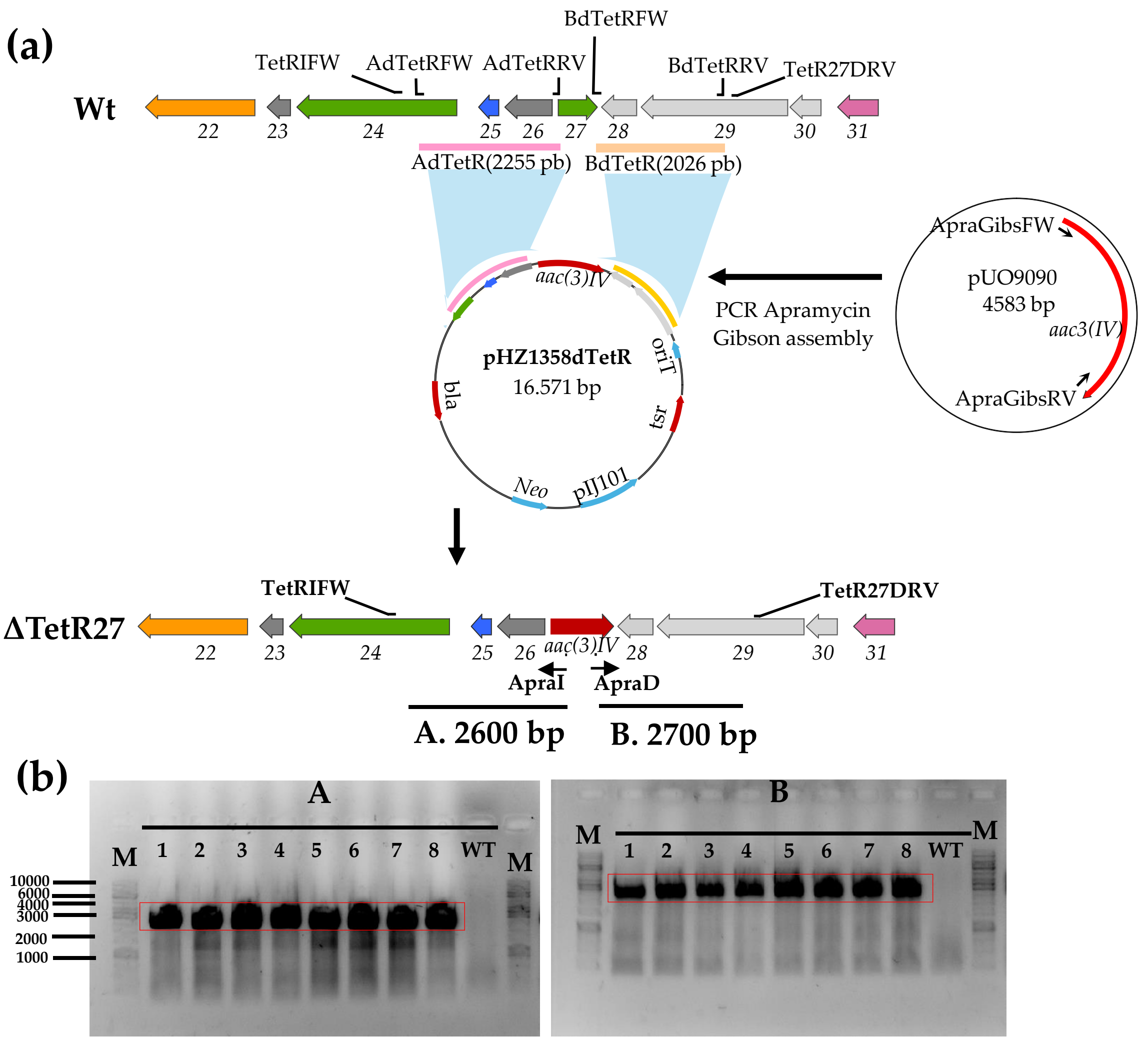

Figure S5. Generation of ΔTetR mutant. (**a**) Scheme representing the construction of pHZ1358ΔTetR by Gibson assembly, and the generation of the ΔTetR mutant by gene replacement of *orf27* (*crxR2*) with the apramycin resistance gene. (**b**) PCR confirmation of ΔTetR using oligonucleotides dTetRIFW and ApraI (A) and oligonucleotides dTetRDRV and ApraD (B). WT, CS057 wild type strain; 1 to 8 correspond to eight independent ΔTetR mutants; *aac3(IV)*, apramycin resistance gene; *bla*, ampicillin resistance gene; *tsrR*, thiostrepton resistance gene; *Neo*: kanamycin resistance gene. M, DNA ladder (Generuler 1 kb).

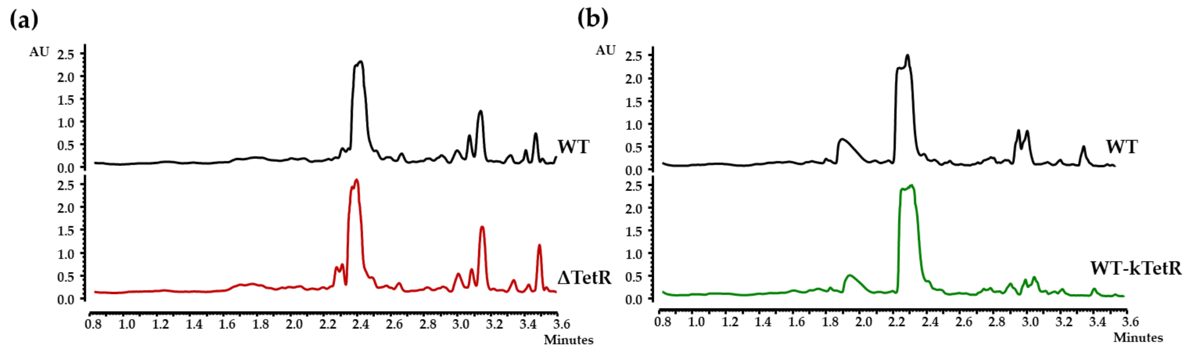

Figure S6. Comparison of metabolite profiles of wild type strain CS057 with ΔTetR (a) and WT-kTetR (b). UPLC chromatograms (Max Plot) of ethyl acetate-1 % formic acid extracts of 5 days-old cultures in R5A medium. WT, wild type strain.

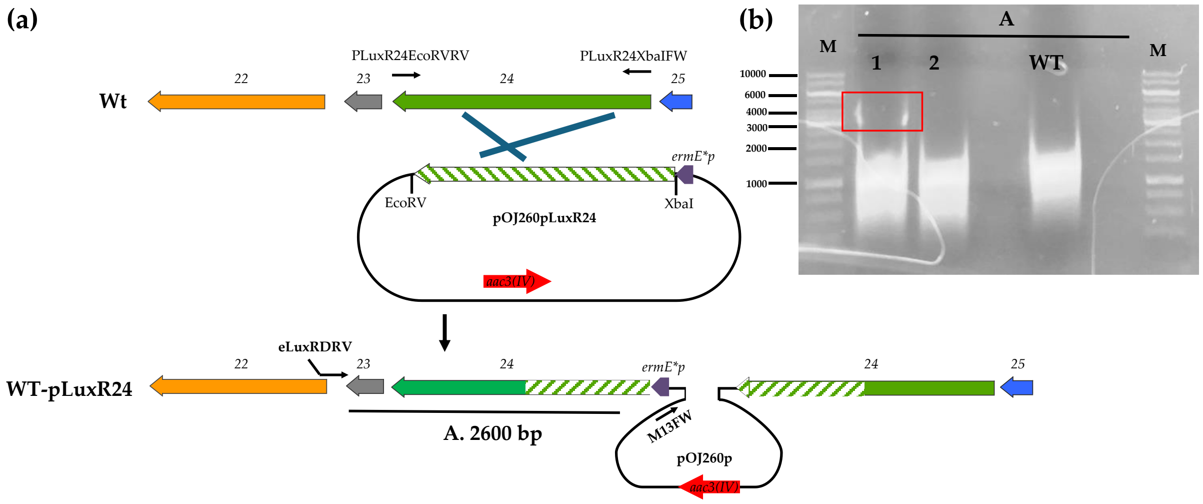

Figure S7. Generation of the WT-pLuxR24 recombinant strain (**a**) Scheme representing the generation of WT-pLuxR24 sttrain. (**b**) PCR confirmation of WT-pLuxR24 using oligonucleotides M13FW and eLuxR24DRV. 1 corresponds to WT-pLuxR24 strain. WT, wild type; *aac3(IV)*, apramycin resistance gene; *ermE*p*, erythromycin resistance promoter; M, DNA ladder (Generuler 1 Kb).

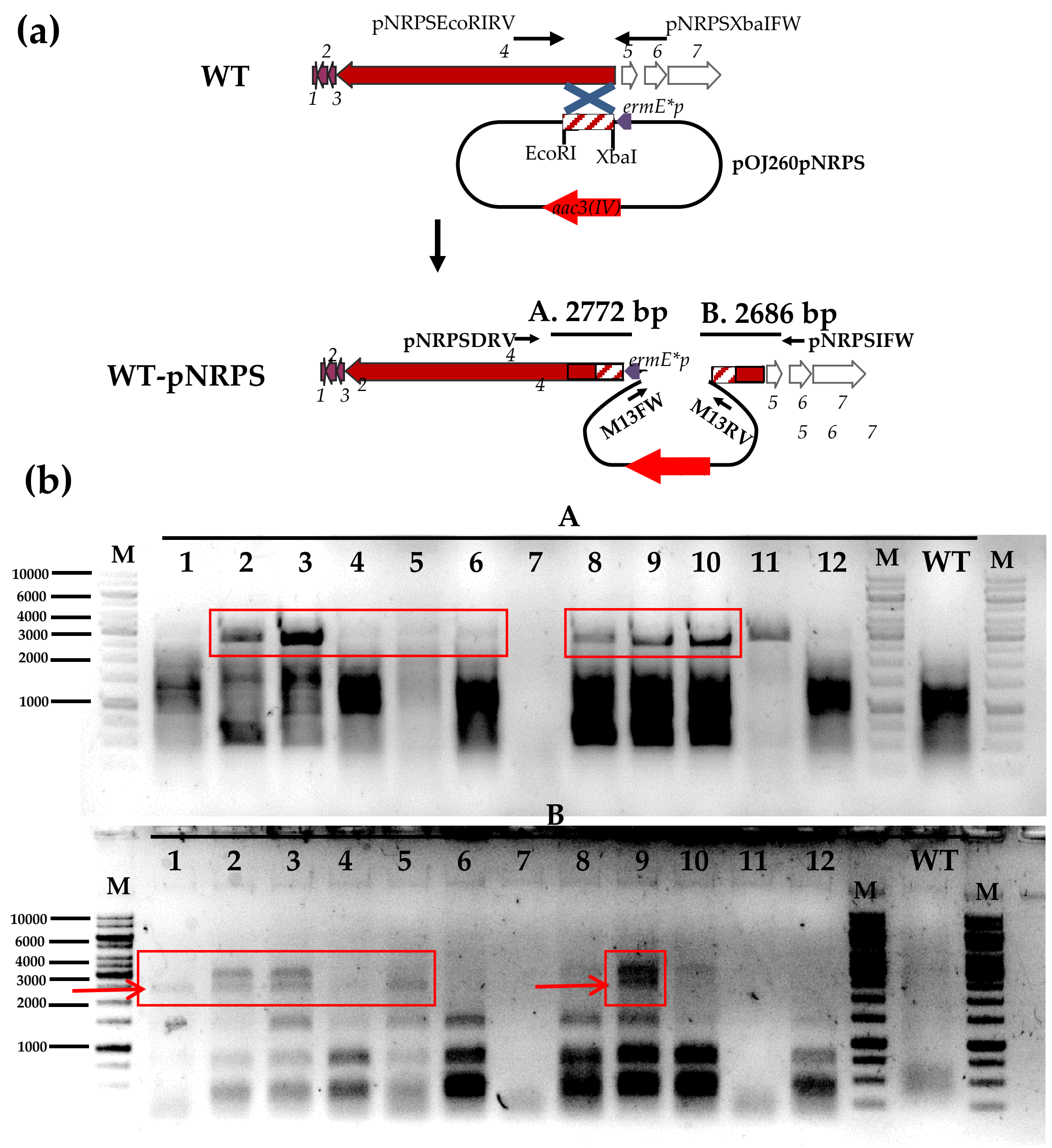

Figure S8. Generation of the WT-pNRPS recombinant strain. (a) Scheme representing the generation of WT-pNRPS. (**c**) PCR confirmation of WT-pNRPS using oligonucleotides pNRPSDRV and M13FW (A); and using oligonucleotides pNRPSIFW and M13RV (B). 2-5 and 9 are five independent WT-pNRPS. WT, wild type; *aac3(IV)*, apramycin resistance gene; *ermE*p*, erythromycin resistance promoter; M, DNA ladder (Generuler 1 Kb).

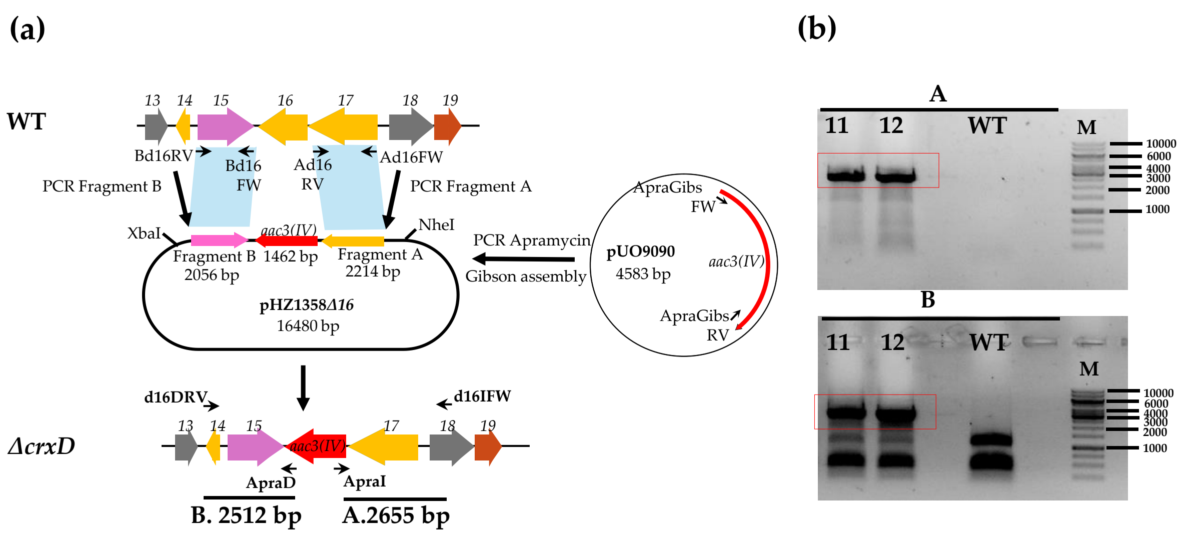

Figure S9. Generation of the Δ*crxD* mutant strain. (**a**) Scheme representing the construction of pHZ1358Δ16 by Gibson assembly, and the generation of the Δ*crxD* mutant by gene replacement of *crxD* (*orf16*) by the apramycin resistance gene. (**b**) PCR confirmation of CS057Δ*crxD* using oligonucleotides d16IFW and ApraI (A) and oligonucleotides d16DRV and ApraD (B). 11 and 12 correspond to two independent Δ*crxD* mutants. WT, wild type strain; *aac3*(IV), apramycin resistance gene; M, DNA ladder (Generuler 1 kb).

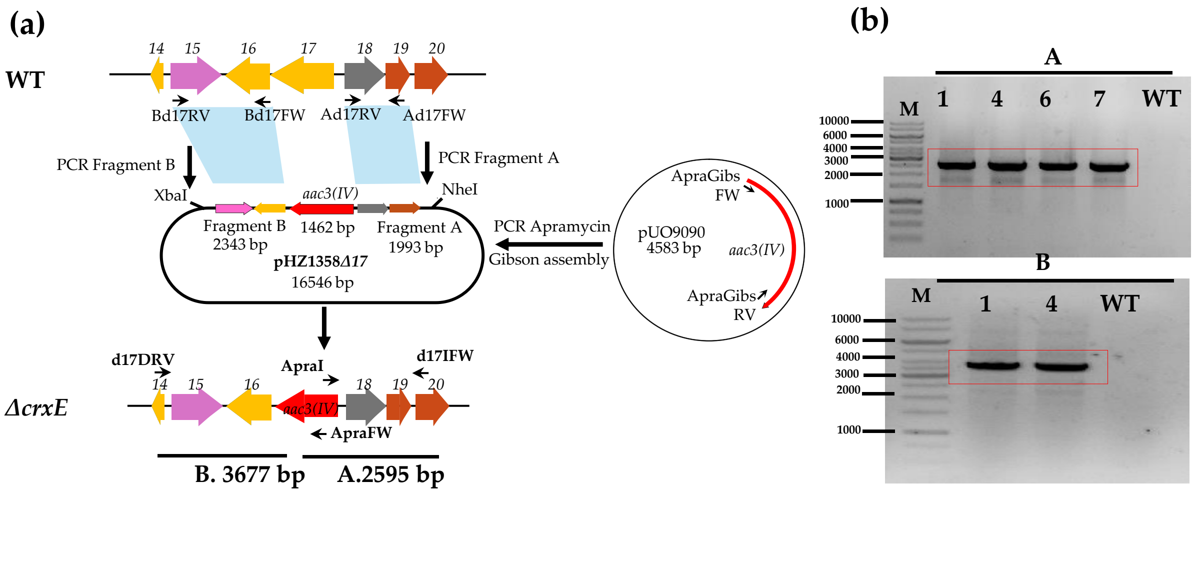

Figure S10. Generation of the Δ*crxE* mutant strain. (**a**) Scheme representing the construction of pHZ1358Δ17 by Gibson assembly, and the generation of the Δ*crxE* mutant by gene replacement of *crxE* (*orf17*) with the apramycin resistance gene. (**b**)

PCR for confirmation of Δ*crxE* using oligonucleotides d17IFW and ApraI (A) and oligonucleotides d17DRV and ApraFW (B). 1 and 4 correspond to four independent Δ*crxE* mutants. WT, wild type strain; *aac3*(IV), apramycin resistance gene; M, DNA ladder (Generuler 1 kb).

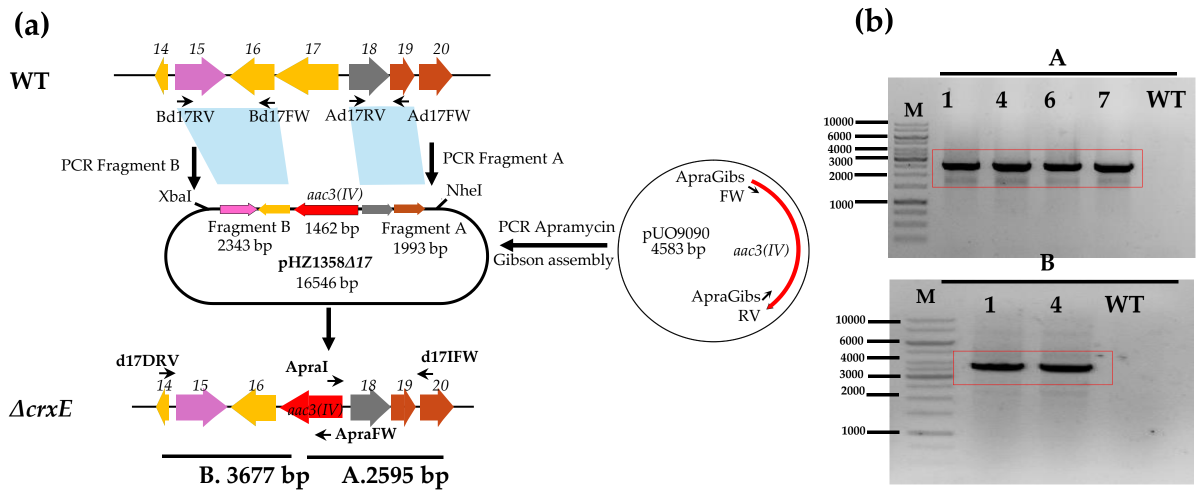

Figure S11. Generation of the Δ*crxM* mutant strain. (**a**) Scheme representing the construction of pHZ1358Δ22 by Gibson assembly, and the generation of the Δ*crxM* mutant by gene replacement of *crxM* (*orf22*) by apramycin resistance gene. (**b**) PCR confirmation of ΔcrxM using oligonucleotides d22IFW and ApraI (A) and oligonucleotides d22DRV and ApraD (B). 3, 4, and 6 correspond to three independent ΔcrxM mutants. WT, wild type strain; *aac3*(IV), apramycin resistance gene; M, DNA ladder (Generuler 1 kb).

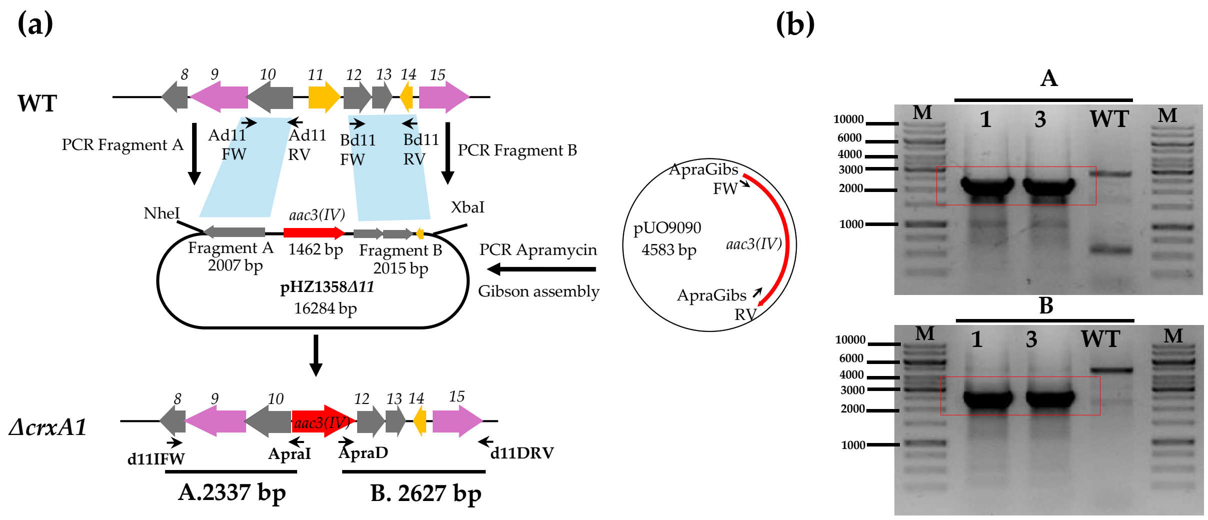

Figure S12. Generation of the Δ*crxA1* mutant strain. (**a**) Scheme representing the construction of pHZ1358Δ11 by Gibson assembly, and the generation of the Δ*crxA1* mutant by gene replacement of *crxA1* (*orf11*) with the apramycin resistance gene. (**b**) PCR confirmation of Δ*crxA1* using oligonucleotides d11IFW and ApraI (A) and oligonucleotides d11DRV and ApraD (B). 1 and 3 correspond to two independent Δ*crxA1* mutants. WT, wild type strain; *aac3*(IV), apramycin resistance gene; M, DNA ladder (Generuler 1 kb).

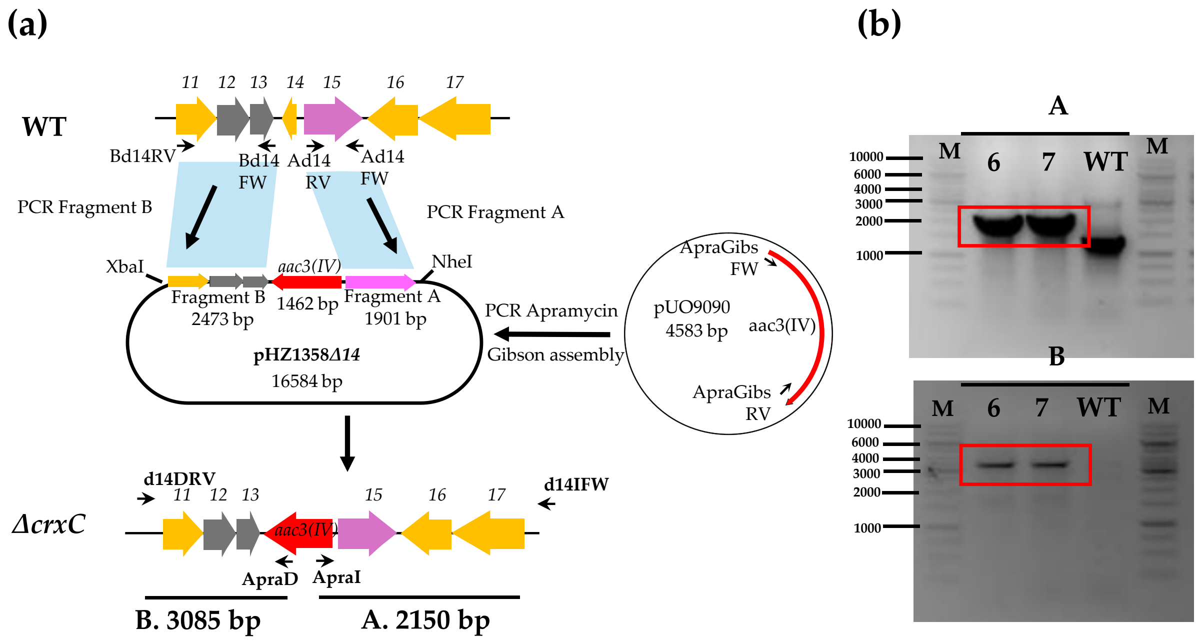

Figure S13. Generation of the Δ*crxC* mutant strain. (**a**) Scheme representing the construction of pHZ1358Δ14 by Gibson assembly, and the generation of the Δ*crxC* mutant by gene replacement of *crxC* (*orf14*) with the apramycin resistance gene. (**b**)

PCR for the confirmation of Δ*crxC* using oligonucleotides d14IFW and ApraI (A), and d14DRV and ApraD (B). 6 and 7 correspond to two independent Δ*crxC* mutants. WT, wild type strain; *aac3*(IV), apramycin resistance gene; M, DNA ladder (Generuler 1 kb).

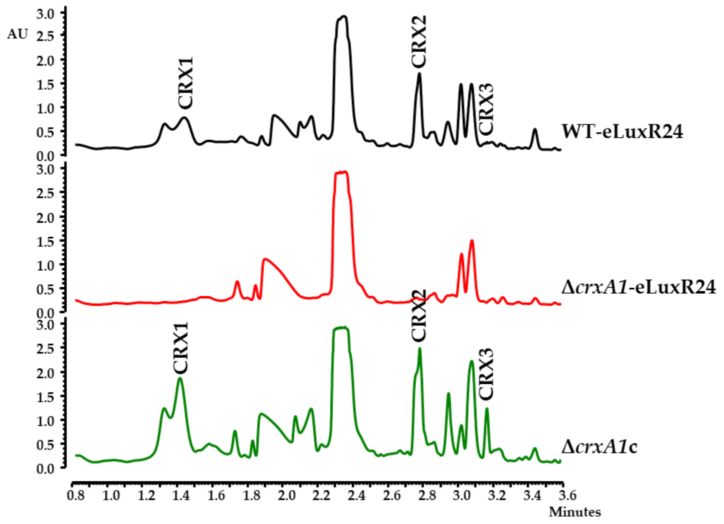

Figure S14. Complementation of ΔcrxA1 mutant. Metabolite profile of ΔcrxA1c complemented mutant in comparison to the Δ*crxA1*-eLuxR24 mutant and the WT-eLuxR24 strain. UPLC chromatograms (Max Plot) of ethyl acetate-1 % formic acid extracts of 5 days-old cultures in R5A medium. WT, wild type strain.

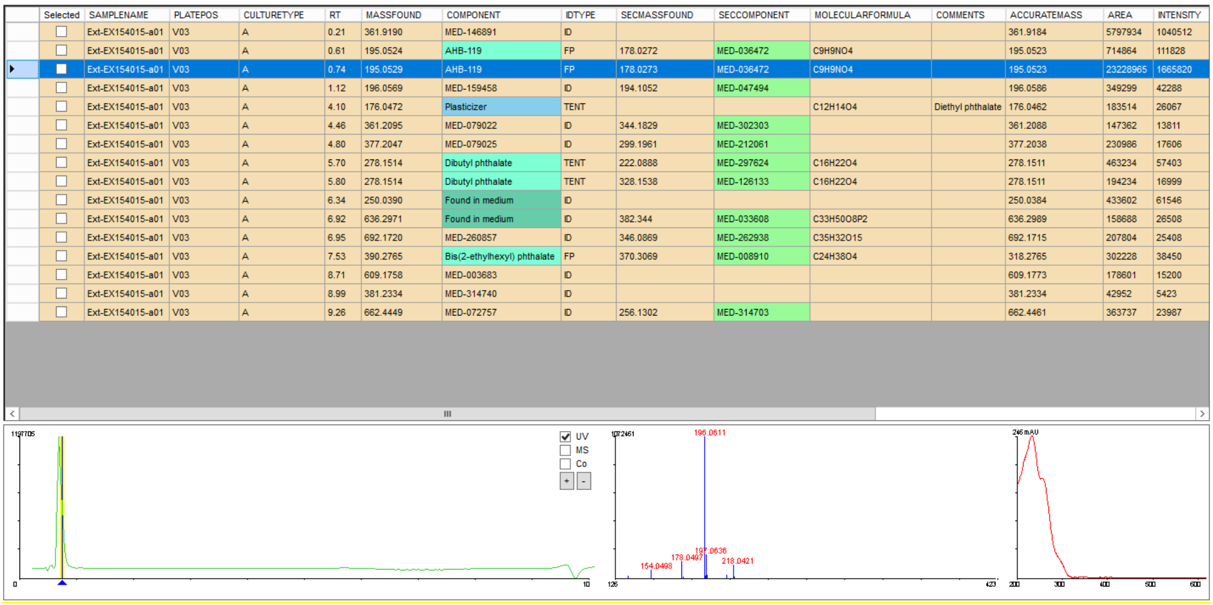

Selected	SAMPLENAME	PLATEPOS	CULTURETYPE	RT	MASSFOUND	COMPONENT	IDTYPE	SECMASSFOUND	SECCOMPONENT	MOLECULARFORMULA	COMMENTS	ACCURATEMASS	AREA	INTENSITY
	Ext-EX154015-a01	V03	A	0.21	361.9190	MED-146891	ID					361.9184	5797934	1040512
	Ext-EX154015-a01	V03	A	0.61	195.0524	AHB-119	FP	178.0272	MED-036472	C9H9NO4		195.0523	714864	111828
	Ext-EX154015-a01	V03	A	0.74	195.0529	AHB-119	FP	178.0273	MED-036472	C9H9NO4		195.0523	23228965	1665820
	Ext-EX154015-a01	V03	A	1.12	196.0569	MED-159458	ID	194.1052	MED-047494			196.0586	349299	42288
	Ext-EX154015-a01	V03	A	4.10	176.0472	Plasticizer	TENT			C12H14O4	Diethyl phthalate	176.0462	183514	26067
	Ext-EX154015-a01	V03	A	4.46	361.2095	MED-079022	ID	344.1829	MED-302303			361.2088	147362	13811
	Ext-EX154015-a01	V03	A	4.80	377.2047	MED-079025	ID	299.1961	MED-212061			377.2038	230986	17606
	Ext-EX154015-a01	V03	A	5.70	278.1514	Dibutyl phthalate	TENT	222.0888	MED-297624	C16H22O4		278.1511	463234	57403
	Ext-EX154015-a01	V03	A	5.80	278.1514	Dibutyl phthalate	TENT	328.1538	MED-126133	C16H22O4		278.1511	194234	16999
	Ext-EX154015-a01	V03	A	6.34	250.0390	Found in medium	ID					250.0384	433602	61546
	Ext-EX154015-a01	V03	A	6.92	636.2971	Found in medium	ID	382.344	MED-033608	C33H50O8P2		636.2989	158688	26508
	Ext-EX154015-a01	V03	A	6.95	692.1720	MED-260857	ID	346.0869	MED-262938	C35H32O15		692.1715	207804	25408
	Ext-EX154015-a01	V03	A	7.53	390.2765	Bis(2-ethylhexyl) phthalate	FP	370.3069	MED-008910	C24H38O4		318.2765	302228	38450
	Ext-EX154015-a01	V03	A	8.71	609.1758	MED-003683	ID					609.1773	178601	15200
	Ext-EX154015-a01	V03	A	8.99	381.2334	MED-314740	ID					381.2334	42952	5423
	Ext-EX154015-a01	V03	A	9.26	662.4449	MED-072757	ID	256.1302	MED-314703			662.4461	363737	23987

Figure S15: Identification of CRX1 by dereplication. Screenshot of our developed dereplication application based on LC-DAD-HRMS analysis and an in-house library (designed as a relational database) showing the identification of CRX1 as *N*-acetyl-3,4-AHBA (also known as ahbamycin AHB119). UV (DAD) and HR-ES(+)-TOF MS spectra of CRX1 are displayed for convenience alongside the chromatogram highlighting its retention time.

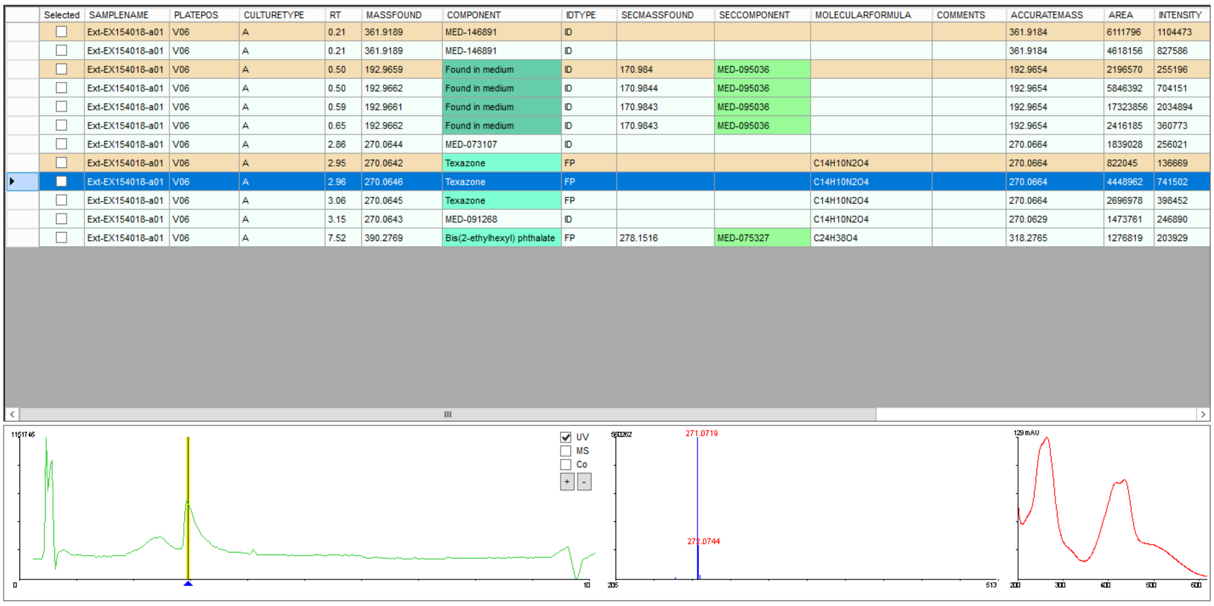

Selected	SAMPLENAME	PLATEPOS	CULTURETYPE	RT	MASSFOUND	COMPONENT	IDTYPE	SECMASSFOUND	SECCOMPONENT	MOLECULARFORMULA	COMMENTS	ACCURATEMASS	AREA	INTENSITY
	Ext-EX154018-a01	V06	A	0.21	361.9189	MED-146891	ID					361.9184	6111796	1104473
	Ext-EX154018-a01	V06	A	0.21	361.9189	MED-146891	ID					361.9184	4618156	827586
	Ext-EX154018-a01	V06	A	0.50	192.9659	Found in medium	ID	170.984	MED-095036			192.9654	2196570	255196
	Ext-EX154018-a01	V06	A	0.50	192.9662	Found in medium	ID	170.9844	MED-095036			192.9654	5846392	704151
	Ext-EX154018-a01	V06	A	0.59	192.9661	Found in medium	ID	170.9843	MED-095036			192.9654	17323856	2034894
	Ext-EX154018-a01	V06	A	0.65	192.9662	Found in medium	ID	170.9843	MED-095036			192.9654	2416185	360773
	Ext-EX154018-a01	V06	A	2.86	270.0644	MED-073107	ID					270.0664	1839028	256021
	Ext-EX154018-a01	V06	A	2.95	270.0642	Texazone	FP			C14H10N2O4		270.0664	822045	136669
	Ext-EX154018-a01	V06	A	2.96	270.0646	Texazone	FP			C14H10N2O4		270.0664	4448962	741502
	Ext-EX154018-a01	V06	A	3.06	270.0645	Texazone	FP			C14H10N2O4		270.0664	2696978	398452
	Ext-EX154018-a01	V06	A	3.15	270.0643	MED-091268	ID			C14H10N2O4		270.0629	1473761	246890
	Ext-EX154018-a01	V06	A	7.52	390.2769	Bis(2-ethylhexyl) phthalate	FP	278.1516	MED-075327	C24H38O4		318.2765	1276819	203929

Figure S16: Identification of CRX3 by dereplication. Screenshot of our developed dereplication application based on LC-DAD-HRMS analysis and an in-house library (designed as a relational database) showing the identification of CRX3 as Texazone.

UV (DAD) and HR-ES(+)-TOF MS spectra of CRX3 are displayed for convenience alongside the chromatogram highlighting its retention time.

a)

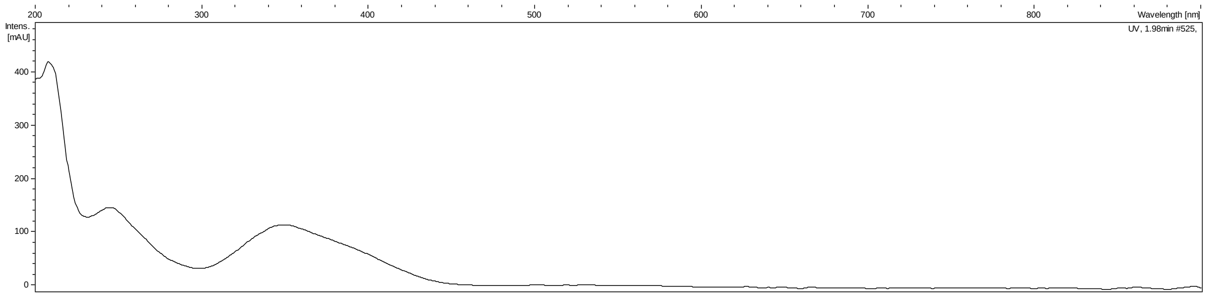

b)

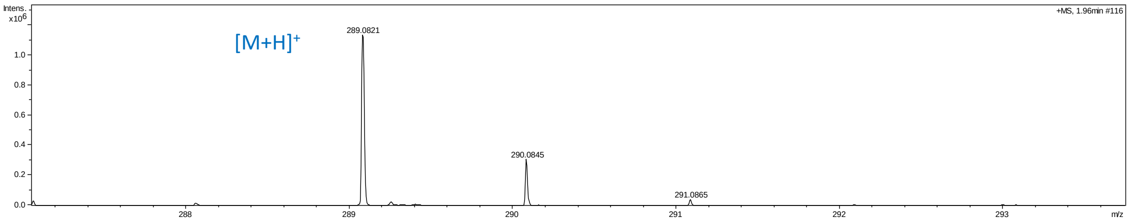

Figure S17: **a)** UV (DAD) spectrum of crexazone 2. **b)** HR-ESI(+)-TOF MS spectrum of CRX2.

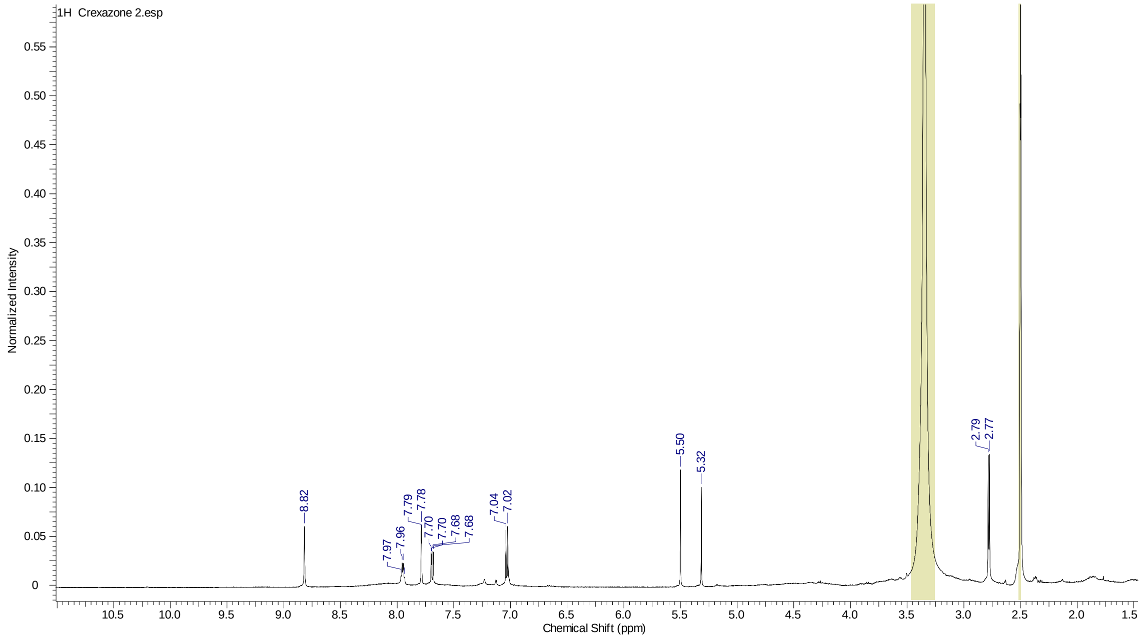

Figure S18: ^{1}H NMR spectrum (DMSO-d_6, 500 MHz, 24 °C) of CRX2.

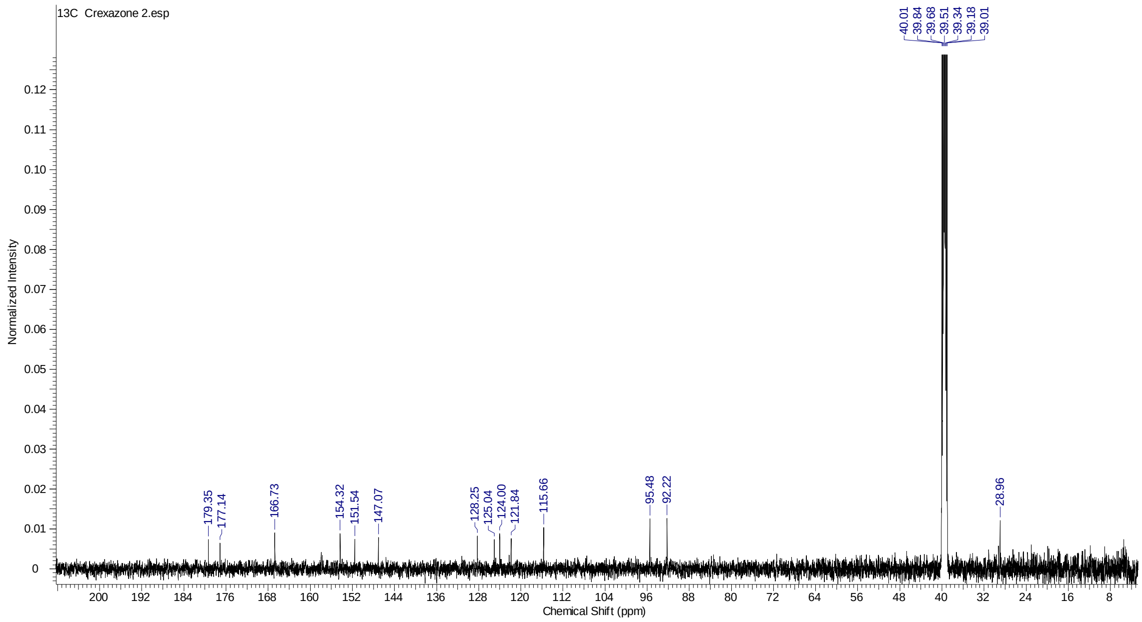

Figure S19: ^{13}C NMR spectrum (DMSO-*d*$_6$, 125 MHz, 24 °C) of CRX2.

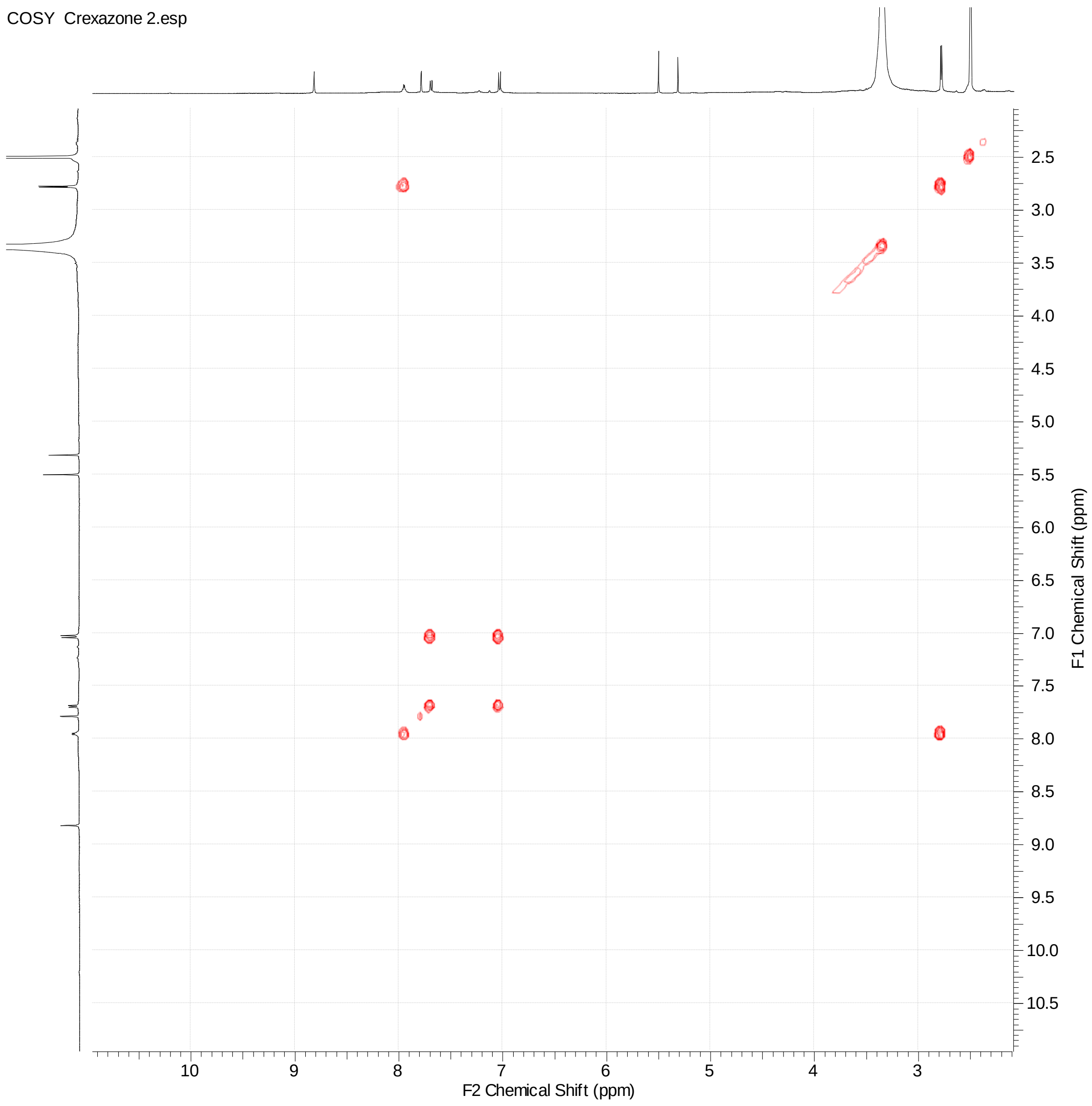

Figure S20: COSY spectrum of CRX2.

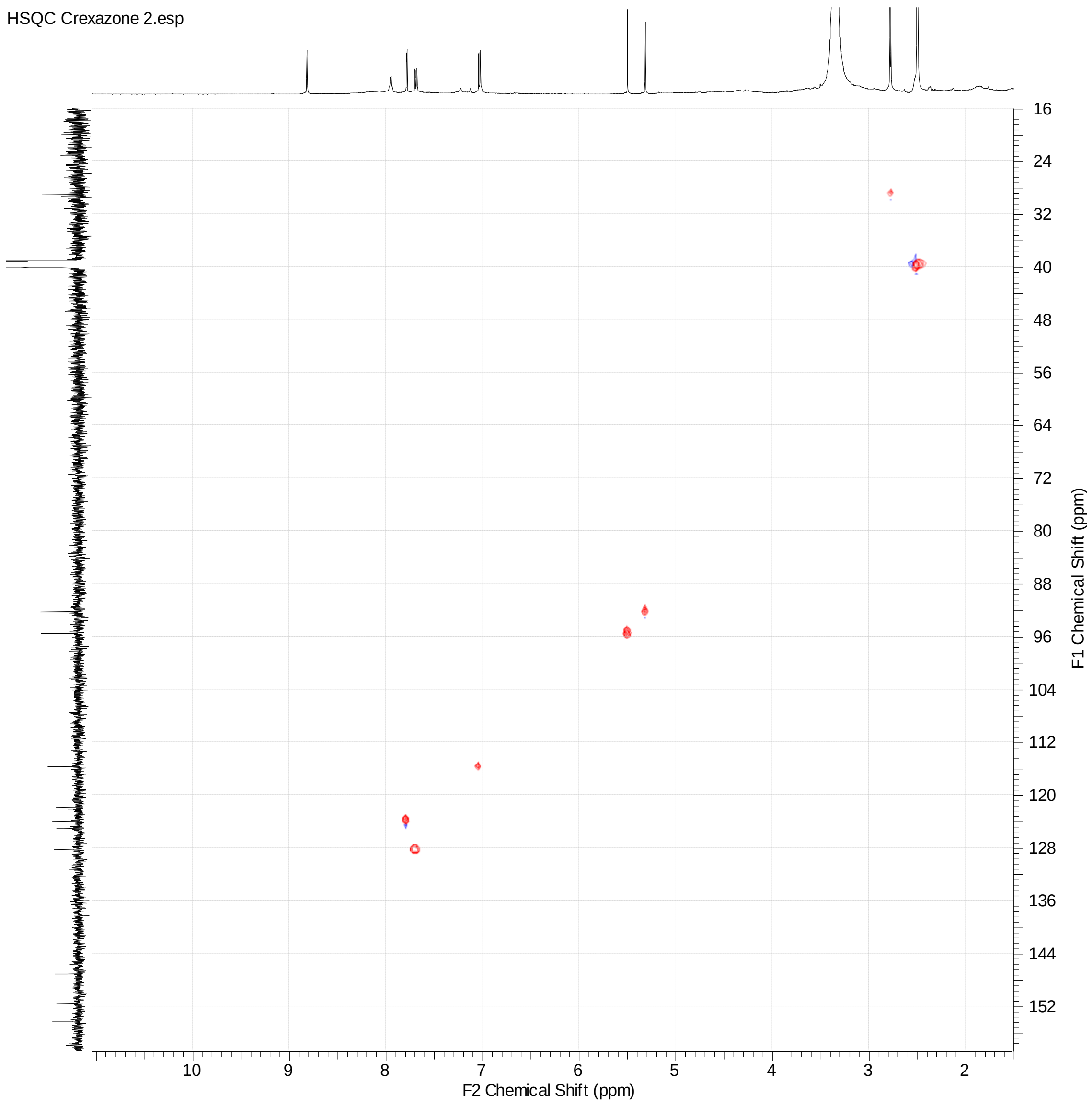

Figure S21: HSQC spectrum of CRX2.

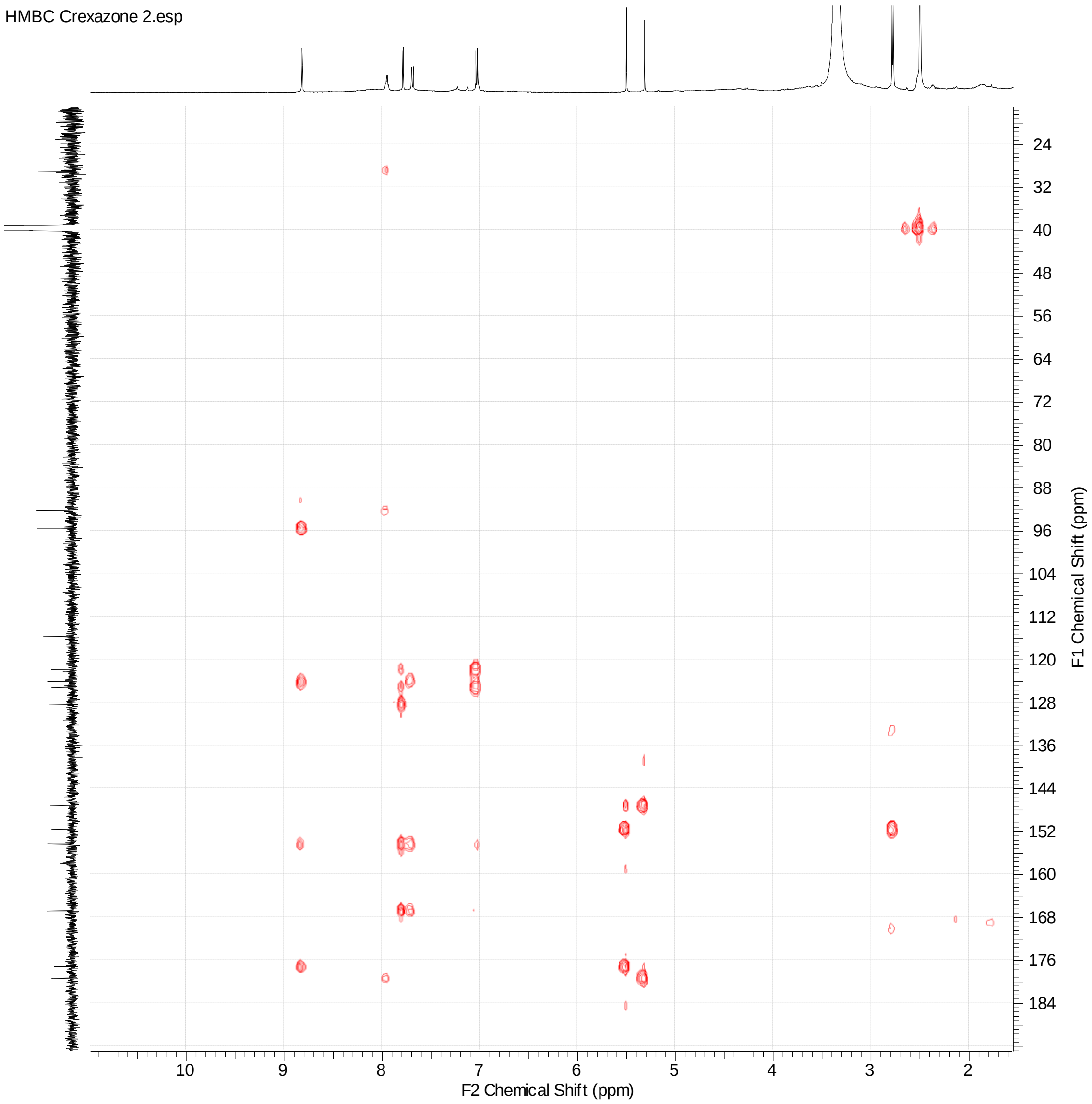

Figure S22: HMBC spectrum of CRX2.

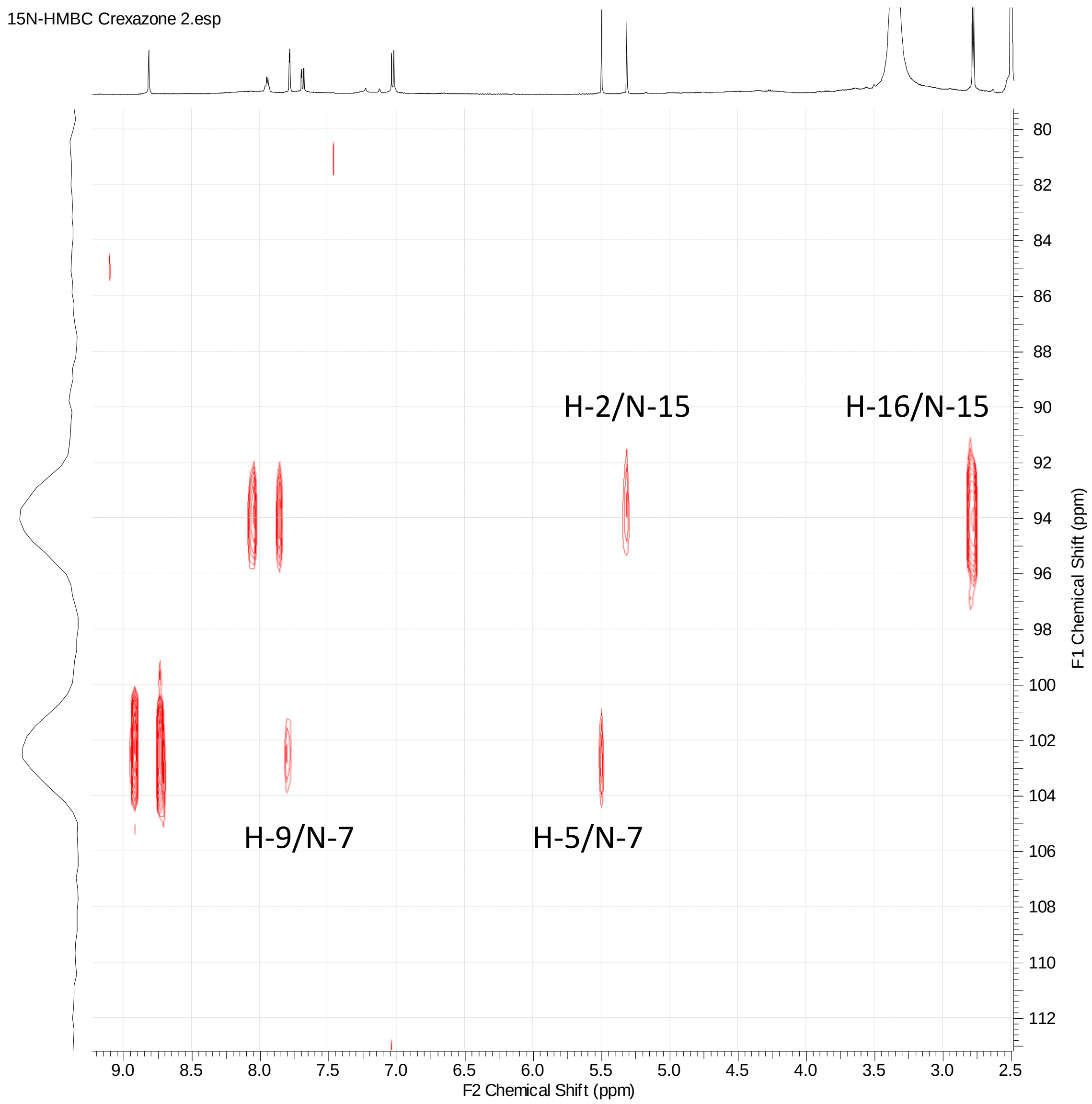

Figure S23: ^{15}N-HMBC spectrum of CRX2 (zoomed region). (Doublet signals correspond to non-filtered one-bond ^{15}N-^{1}H correlati
